# The relative efficiency of staircase and stepped wedge cluster randomised trial designs

**DOI:** 10.1177/09622802251317613

**Published:** 2025-02-16

**Authors:** Kelsey L Grantham, Andrew B Forbes, Richard Hooper, Jessica Kasza

**Affiliations:** 1 School of Public Health and Preventive Medicine, Monash University, Melbourne, VIC, Australia; 2 Wolfson Institute of Population Health, 4617 Queen Mary University of London, London, UK

**Keywords:** Clinical trial design, incomplete design, intracluster correlation, sample size, trial planning

## Abstract

The stepped wedge design is an appealing longitudinal cluster randomised trial design. However, it places a large burden on participating clusters by requiring all clusters to collect data in all periods of the trial. The staircase design may be a desirable alternative: treatment sequences consist of a limited number of measurement periods before and after the implementation of the intervention. In this article, we explore the relative efficiency of the stepped wedge design to several variants of the ‘basic staircase’ design, which has one control followed by one intervention period in each sequence. We model outcomes using linear mixed models and consider a sampling scheme where each participant is measured once. We first consider a basic staircase design embedded within the stepped wedge design, then basic staircase designs with either more clusters or larger cluster-period sizes, with the same total number of participants and with fewer total participants than the stepped wedge design. The relative efficiency of the designs depends on the intracluster correlation structure, correlation parameters and the trial configuration, including the number of sequences and cluster-period size. For a wide range of realistic trial settings, a basic staircase design will deliver greater statistical power than a stepped wedge design with the same number of participants, and in some cases, with even fewer total participants.

## 1 Introduction

**Figure 1.**
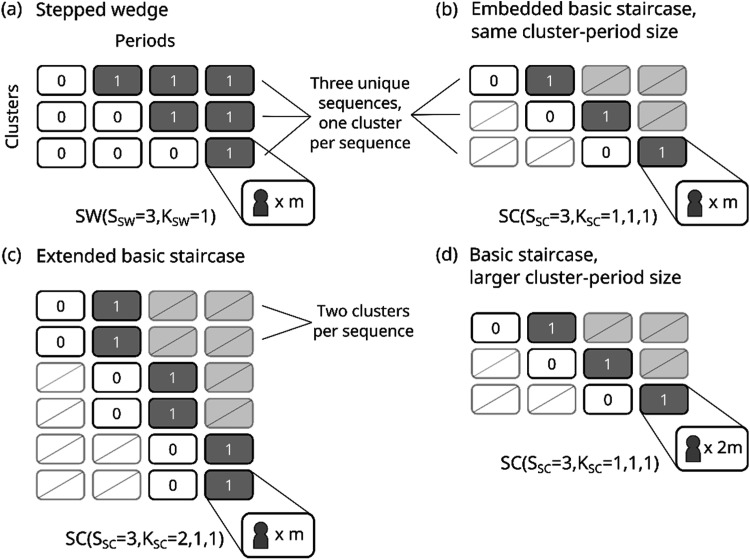
Design schematics for three-sequence designs, shown with a fixed number of clusters per sequence for illustrative purposes: (a) a stepped wedge design with one cluster per sequence and a cluster-period size of m , (b) an embedded basic staircase design with one cluster per sequence and a cluster-period size of m , (c) an extended basic staircase design with two clusters per sequence and a cluster-period size of m , and (d) a basic staircase design with one cluster per sequence and a cluster-period size of 2m . Cells contain either 0 for the control condition, 1 for the intervention condition, or a slash to indicate that no measurements are taken.

The stepped wedge cluster randomised trial design, which randomises the order in which clusters of participants commence implementation of the intervention, is an appealing yet potentially burdensome design. In typical applications of the design, all clusters start out implementing the control condition and end up implementing the intervention condition, with the timing of the switch from control to intervention being staggered over the trial periods. The design schematic resembles two triangular wedges of control and intervention cells, with a zigzag of steps along the diagonal where the switches occur (Figure 1(a)). This design is particularly appealing because it allows the intervention to be gradually rolled out across the participating clusters and all clusters will eventually receive the intervention.^1,2^ In a stepped wedge design, all participating clusters collect and provide data in each period of the trial (i.e. in each ‘cluster-period’ cell of the design). Extending data collection over multiple time periods has advantages: the opportunity to collect more data from each cluster, and to switch a cluster from control to intervention so that it acts as its own control, for example. But even in this case, it may not be necessary to collect data from every cluster in every period – indeed, this may be costly or burdensome. For example, a recent study investigating the integration of palliative care into residential aged care facilities in Australia noted the high burden of data collection in a stepped wedge design.^3^ In this study, data collection for several outcomes of interest involved completing questionnaires which would have taken large amounts of staff time to complete for each participant throughout the duration of the trial; the researchers described the need to limit the length and number of measures due to the burden data collection would place on staff. Thus, there is a need to investigate alternative, less burdensome designs.

Recent investigations have revealed that not all cluster-period cells in a stepped wedge design contribute equally to the estimation of the treatment effect, and have also considered how we might plan data collection in a stepped wedge trial in a more efficient way. Kasza and Forbes^4^ showed that some periods of measurement in stepped wedge designs are much more informative about the treatment effect than others. Specifically, measurements from clusters in periods just before and after the switch from the control to the intervention conditions are more ‘information-rich’ than many cluster-period cells further away from the steps. In addition, cells in the corners of the design schematic may also contribute a relatively large amount of information, depending on the underlying modelling assumptions. More recently, Rezaei-Darzi et al.^5^ showed that when low-information cluster-period cells were iteratively removed from a complete stepped wedge design to obtain a series of progressively more ‘incomplete’ designs, designs containing only cluster-period cells around the time of the switch from control to intervention in each sequence often retained adequate power. Similar patterns have emerged in the study of stepped wedge designs where participants are recruited in continuous time.^6^ While these studies have considered linear mixed models, cells around the time of the treatment switch also appear to be the most information-rich when marginal models are fit via generalised estimating equations.^7^ These findings motivate the further study of incomplete stepped wedge designs, where clusters are not required to provide data in all periods of the trial. In certain situations, incomplete designs such as the staircase design may provide sufficient power for testing new interventions.

The staircase design is a longitudinal cluster randomised trial design with treatment sequences containing a limited number of control periods followed by a limited number of intervention periods, where these measurement periods are staggered over time (e.g. Figure 1(b)).^8^ Staircase designs are already being conducted in practice despite limited investigations into the statistical properties of these designs.^9–13^ A few recent studies have examined the efficiency of incomplete relative to complete stepped wedge designs for particular trial configurations, with staircase-like designs among the incomplete designs considered.^5,14,15^ Unni et al.^14^ compared the power of three incomplete designs (a stepped wedge design with implementation periods, a staircase design with sequences consisting of one control period and two intervention periods, and a batched stepped wedge design), to a complete stepped wedge design in the context of a trial targeting heart failure. Kasza et al.^15^ considered the power of four incomplete stepped wedge designs in a different trial context, where the incomplete designs were chosen based on the patterns of information contributed by cluster-period cells for the particular trial example. These designs resembled staircase designs with or without cluster-periods in the corners of the design schematic, including a staircase design with one control period followed by one intervention period in each sequence, and a staircase design with sequences containing a mix of two or three measurement periods around the main diagonal. These studies found that staircase-like designs had notably less power than the complete stepped wedge design, though this is not surprising as the considered incomplete designs simply deleted cluster-period cells from the complete stepped wedge design and therefore included considerably fewer total participants. Staircase-like designs showed more promise by Rezaei-Darzi et al.,^5^ where they were shown to have low precision loss compared to the complete stepped wedge design in certain trial contexts. An enhanced understanding of staircase designs and how their statistical properties compare to those of stepped wedge designs more generally is imperative.

The set of all possible staircase designs is large, so here we focus on a subclass called ‘basic staircase’ designs.^8^ A basic staircase design has just one pre- and one post-switch measurement period in each sequence, with each sequence commencing data collection in a different trial period, forming a zigzag of steps as shown in Figure 1(b) to (d). To get a more complete understanding of when a staircase design might be chosen on efficiency grounds, we will consider several variants of the basic staircase design. Figure 1(b) to (d) depicts example design schematics of these variants. As our measure of efficiency, we will consider the variance (or alternatively, the precision) of the treatment effect estimator, where here we consider the generalised least squares estimator from linear mixed models, as is commonly considered in the design of stepped wedge trials. We will consider continuous outcomes but note that linear mixed models are sometimes also assumed for binary outcomes, for example, Hemming et al.^16^ We will evaluate the efficiency of several basic staircase designs relative to the stepped wedge design for a range of realistic trial configuration parameters. First, we aim to determine the situations where a basic staircase design (e.g. Figure 1(b)) can be nearly as efficient as the stepped wedge design from which it was derived (e.g. Figure 1(a)), acknowledging that with other parameters fixed including cluster-period size, the staircase design will always be less efficient simply because it necessarily has fewer participants. We then will consider basic staircase designs with additional clusters assigned to each sequence (e.g. Figure 1(c)) and basic staircase designs with larger cluster-period sizes (e.g. Figure 1(d)), that have the same total number of participants as their comparator stepped wedge designs, and that have fewer total participants. In situations where there is some flexibility in the number of participating clusters or the number of participants that can be measured in each cluster-period, our central question is whether a staircase design can be as precise or more precise than a stepped wedge design.

The article is organised as follows: in Section 2, we introduce the design notation, statistical model, expressions for the variance of the treatment effect estimator for stepped wedge and staircase designs, and the relative efficiency metric. In Section 3, we then assess the relative efficiency of the stepped wedge design compared to different variants of the basic staircase design, with one control period followed by one intervention period in each sequence. We consider a stepped wedge trial inspired by a real trial in Section 4, comparing the efficiency of this design to those reimagined as staircase designs, and provide some concluding remarks in Section 5.

## 2 Statistical model and variance expressions

### 2.1 Design characteristics and notation

We first consider standard stepped wedge designs in which all sequences implement the control condition in the first period and the intervention condition in the last period, and each sequence switches over to the intervention in a different intermediate period of the trial. We denote such stepped wedge (SW) designs by $SW(S_{SW},K_{SW})$ , where $S_{SW}$ is the number of unique treatment sequences spanning $S_{SW}+1$ periods and $K_{SW}$ is the number of clusters assigned to each sequence. We then consider basic staircase (SC) designs as by Grantham et al.^8^ denoted by $SC(S_{SC},K_{SC},R_{0},R_{1})$ with $S_{SC}$ unique treatment sequences spanning a total of $S_{SC}+1$ periods with $K_{SC}$ clusters assigned to each sequence, where each sequence consists of one pre-switch control period $\left(R_{0}=1\right)$ followed by one post-switch intervention period $\left(R_{1}=1\right)$ and sequence *s* begins taking measurements in period *s*, $s=1,\ldots ,S_{SC}$ . We consider trial settings where each participant is measured just once and different participants enter the cluster in each period. We will address extensions to cohort designs, where participants may be measured more than once over the course of the trial, and to designs with implementation periods, in the Discussion.

### 2.2 Statistical model for continuous outcomes

We assume a linear mixed model for continuous outcomes appropriate for longitudinal cluster randomised trial designs, with special cases of stepped wedge (with $K=K_{SW}$ and $S=S_{SW}$ ) and staircase (with $K=K_{SC}$ and $S=S_{SC}$ ) designs, where the measured outcome $Y_{skti}$ for participant i=1,…,m in cluster k=1,…,K assigned to sequence s=1,…,S in period $t=t_{1},\ldots ,t_{J}$ (where $t_{1}=1$ and $t_{J}=S+1$ for stepped wedge designs and $t_{1}=s$ and $t_{J}=s+1$ for basic staircase designs) is given by the following equation:

(1) $$\begin{array}{rl} Y_{skti} & =Z_{st}\beta +X_{st}\theta +CP_{skt}+\epsilon_{skti},\epsilon_{skti}∼N(0,\sigma_{\epsilon }^{2})\\ CP_{sk} & =(CP_{skt_{1}},\ldots ,CP_{skt_{J}}{)}^{T}∼N_{J}(0,V_{CP}) \end{array}$$

where β is a column vector of fixed time effects; $Z_{st}$ is a row vector specifying the form for the fixed effects corresponding to sequence *s* in period *t* (in Section 3, we will assume categorical period effects where $\beta =(\beta_{1},\ldots ,\beta_{S+1}{)}^{T}$ and $Z_{st}$ is an (S+1) -dimensional vector comprised of a 1 in the t th position and zeros elsewhere, and we will touch on results for linear period effects where $\beta =(\beta_{1},\beta_{2}{)}^{T}$ and $Z_{st}=(1,t)$ in the Discussion); $X_{st}$ is the treatment indicator for sequence *s* in period *t*; θ is the treatment effect; $CP_{skt}$ is the cluster-period random effect corresponding to the *k*th cluster assigned to sequence *s* in period *t*; and $\epsilon_{skti}$ is the participant-level random error term; additional covariates could also be included in this model.

While $V_{CP}$ can take one of many possible forms, as by Grantham et al.^8^ we will focus on two intracluster correlation (ICC) structures that account for a decay in correlation between participants’ outcomes measured in different periods in the same cluster: the block-exchangeable and discrete-time decay correlation structures. These structures suppose that the $\left(t,t^{\prime }\right)$ th element of $V_{CP}$ is $cov(CP_{skt},CP_{skt^{\prime }})={\sigma_{C}}^{2}r_{tt^{\prime }}$ , 0≤r≤1 , so that $corr(Y_{skti},Y_{skt^{\prime }i^{\prime }})=[{\sigma_{C}}^{2}/({\sigma_{C}}^{2}+{\sigma_{\epsilon }}^{2})]r_{tt^{\prime }}=\rho r_{tt^{\prime }}$ with $r_{tt^{\prime }}=r$ for all pairs $t\neq t^{\prime }$ under the block-exchangeable structure^17,18^ and $r_{tt^{\prime }}=r^{\left|t-t^{\prime }\right|}$ for all $t,t^{\prime }$ under the discrete-time decay correlation structure.^19^ The block-exchangeable and discrete-time decay correlation structures both reduce to the simple exchangeable correlation structure when r=1 , in which there is no decay in correlation over the trial periods.^20^ We will refer to ρ as the within-period ICC and *r* as the cluster autocorrelation,^19^ and will present results for the block-exchangeable correlation structure in Section 3 and discuss results for discrete-time decay correlation in Section 5.

Similarly to Hooper et al.,^17^ Girling and Hemming^18^ and Kasza et al.,^19^ we work with cluster-period averages, that is, model (1) averaged over the *m* participants’ outcomes within each cluster-period:

(2) $$\begin{array}{rl} {\bar{Y}}_{skt} & =Z_{st}\beta +X_{st}\theta +CP_{skt}+{\bar{\epsilon }}_{skt},{\bar{\epsilon }}_{skt}∼N\left(0,\frac{\sigma_{\epsilon }^{2}}{m}\right)\\ CP_{sk} & =(CP_{skt_{1}},\ldots ,CP_{skt_{J}}{)}^{T}∼N_{J}(0,V_{CP}) \end{array}$$

The covariance matrix for a cluster at the cluster-period mean level, assumed common across clusters, will be denoted by $V_{*}$ and takes the form: $V_{*}=V_{CP}+(\sigma_{\epsilon }^{2}/m)I_{J}$ , where $I_{J}$ is the J×J identity matrix. Then assuming without loss of generality that the total variance $\sigma_{C}^{2}+\sigma_{\epsilon }^{2}=1$ , we can represent the variance of a cluster-period mean as $var({\bar{Y}}_{skt})=\sigma_{C}^{2}+\sigma_{\epsilon }^{2}/m=[1+(m-1)\rho ]/m:=a$ and the covariance between cluster-period means as $cov({\bar{Y}}_{skt},{\bar{Y}}_{skt^{\prime }})=\rho r_{tt^{\prime }}:=b$ . For the block-exchangeable correlation structure where $r_{tt^{\prime }}=r$ , we can also represent the correlation between cluster-period means as follows:

(3) $$\begin{array}{r} \psi =corr({\bar{Y}}_{skt},{\bar{Y}}_{skt^{\prime }})=\frac{b}{a}=\frac{m\rho r}{1+(m-1)\rho } \end{array}$$

**Figure 2.**
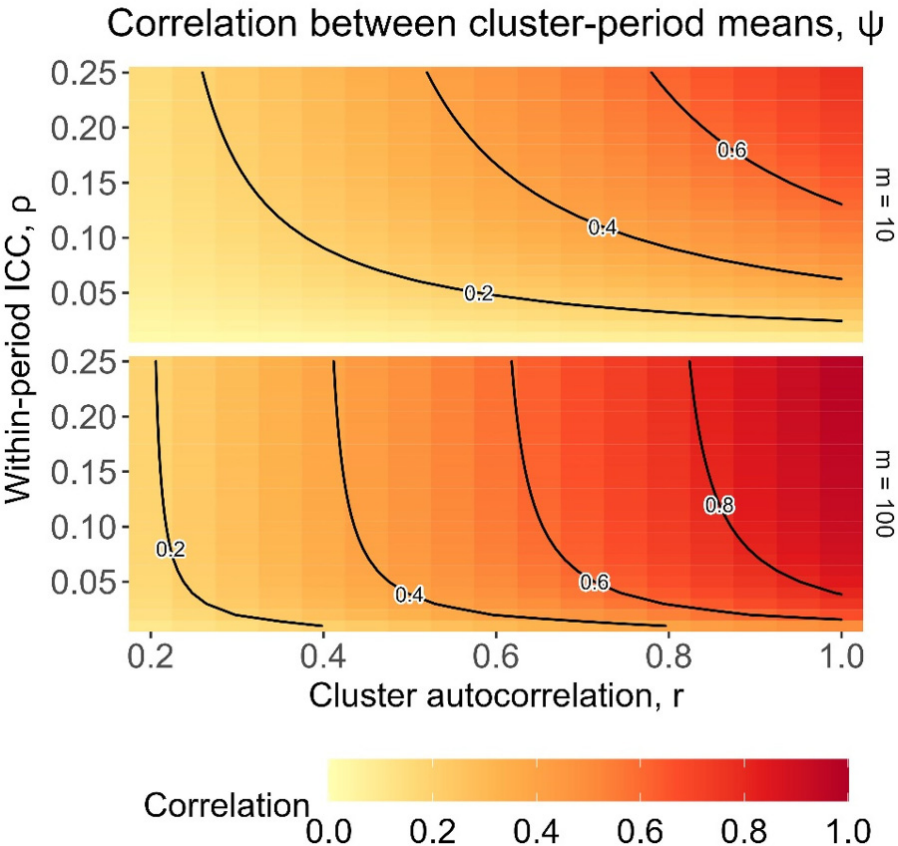
Correlation between cluster-period means under a block-exchangeable correlation structure, for cluster-period sizes of m=10 (top panel) and m=100 (bottom panel), for different combinations of intracluster correlation values.

The parameter ψ is useful as it encodes information about the cluster-period size, within-period ICC, and cluster autocorrelation. Figure 2 displays the values that ψ takes for cluster-period sizes m=10 (top panel) and m=100 (bottom panel), and for ranges of correlation parameters ρ and *r* likely to be seen in practice.^21^ As the cluster-period size *m* increases, the value of ψ approaches the value of *r*. The lowest values of ψ arise when the within-period ICC ρ is very small and/or the cluster autocorrelation *r* is small; larger values of ψ arise for larger values of *r*, together with larger values of ρ , although the value of ρ has less influence for larger cluster-period sizes. The value of ψ approaches the limiting value of 1 with large cluster-period sizes and when the correlation structure approaches that of exchangeable.

### 2.3 Variance of the treatment effect estimator for stepped wedge designs

In this section, we provide expressions for the variance of the treatment effect estimator for the stepped wedge design under a variety of correlation structures. The following section provides analogous variance expressions for staircase designs, and Section 3 directly compares these variance expressions.

Under assumptions of either categorical period effects or a linear time effect over the trial periods, the variance of the treatment effect estimator obtained via generalised least squares appropriate for stepped wedge designs with $S_{SW}$ unique treatment sequences and $K_{SW}$ clusters assigned to each sequence can be represented by the following equation^22^:

(4) $$\begin{array}{r} var(\hat{\theta }{)}_{SW(S_{SW},K_{SW})}=\frac{1}{K_{SW}}{\left[\overset{S_{SW}}{\underset{s=1}{\sum }}\;{X_{s}}^{T}{V_{*}}^{-1}X_{s}-\frac{1}{S_{SW}}{\left(\overset{S_{SW}}{\underset{s=1}{\sum }}\;X_{s}\right)}^{T}{V_{*}}^{-1}\left(\overset{S_{SW}}{\underset{s=1}{\sum }}\;X_{s}\right)\right]}^{-1} \end{array}$$

where $X_{s}=(X_{s1},\ldots ,X_{s,S_{SW}+1}{)}^{T}$ is the *s*th treatment sequence, consisting of *s* zeros followed by $S_{SW}+1-s$ ones, and $V_{*}$ is the covariance matrix for a cluster, assumed to be identical across clusters. We show in Section A.2 of the Supplemental Material that, under a block-exchangeable correlation structure, expression (4) reduces to the following explicit representation:

(5) $$\begin{array}{r} var(\hat{\theta }{)}_{SW(S_{SW},K_{SW})}=\frac{12a(1-\psi )\left(1+S_{SW}\psi \right)}{K_{SW}\left({S_{SW}}^{2}-1\right)\left(2+S_{SW}\psi \right)} \end{array}$$

This expression is equivalent to the expression for the variance of the best linear unbiased estimator of the treatment effect obtained from equation 3 by Girling and Hemming,^18^ the expression for the variance of the treatment effect estimator obtained from the components in Table 1 of Hemming et al.,^16^ and the variance expression under exchangeable correlation by Hussey and Hughes.^20^

### 2.4 Variance of the treatment effect estimator for staircase designs

The variance of the treatment effect estimator obtained via generalised least squares for staircase designs differs from that for stepped wedge designs: the measured cells in all treatment sequences have the same pattern of control and intervention periods (i.e. $X_{s}=X$ ), and the matrices of time effects, $Z_{s}$ , are sequence-dependent such that the variance expression does not simplify to the same extent as in expression (4). The analogue to expression (4) appropriate for staircase designs^8^ is given by the following equation:

(6) $$\begin{array}{r} var(\hat{\theta }{)}_{SC(S_{SC},K_{SC},R_{0},R_{1})}=\frac{1}{S_{SC}K_{SC}}{\left[X^{T}{V_{*}}^{-1}X-\frac{1}{S_{SC}}X^{T}{V_{*}}^{-1}\overset{S_{SC}}{\underset{s=1}{\sum }}\;Z_{s}{\left(\overset{S_{SC}}{\underset{s=1}{\sum }}\;{Z_{s}}^{T}{V_{*}}^{-1}Z_{s}\right)}^{-1}\overset{S_{SC}}{\underset{s=1}{\sum }}\;{Z_{s}}^{T}{V_{*}}^{-1}X\right]}^{-1} \end{array}$$

Explicit expressions for the variance of the treatment effect estimator are also available for basic staircase designs with one period pre- and one period post-intervention,^8^ for which $X=(0,1{)}^{T}$ and hence the block-exchangeable and discrete-time decay correlation structures coincide with a common 2×2 covariance matrix, $V_{*}$ . These expressions, under assumptions of categorical and linear period effects, are given by the following equations:

(7) $$\begin{array}{rl} var(\hat{\theta }{)}_{SC(S_{SC},K_{SC},1,1),cat} & =\frac{2a{\left(1-\psi \right)}^{2}}{K_{SC}\left[S_{SC}(1-\psi )-\sqrt{1-\psi^{2}}\frac{{\left(1+\sqrt{1-\psi^{2}}\right)}^{S_{SC}}-\psi^{S_{SC}}}{{\left(1+\sqrt{1-\psi^{2}}\right)}^{S_{SC}}+\psi^{S_{SC}}}\right]} \end{array}$$

(8) $$\begin{array}{rl} var(\hat{\theta }{)}_{SC(S_{SC},K_{SC},1,1),lin} & =\frac{2a\left[\left(S_{SC}^{2}+2\right)-\left(S_{SC}^{2}-4\right)\psi \right]}{K_{SC}S_{SC}\left(S_{SC}^{2}-1\right)} \end{array}$$

### 2.5 Relative efficiency metric

We define the relative efficiency as the ratio of the precision of the treatment effect estimator for the basic staircase design to the precision for the stepped wedge design as follows:

$$RE=\frac{1/var{\left(\hat{\theta }\right)}_{SC(S_{SC},K_{SC},1,1)}}{1/var{\left(\hat{\theta }\right)}_{SW(S_{SW},K_{SW})}}=\frac{var{\left(\hat{\theta }\right)}_{SW(S_{SW},K_{SW})}}{var{\left(\hat{\theta }\right)}_{SC(S_{SC},K_{SC},1,1)}}$$

Thus, RE>1 indicates that the staircase design leads to a more precise treatment effect estimator than the stepped wedge design. While the variance expressions from Sections 2.3 and 2.4 depend on *a*, ψ , *S*, and *K*, the relative efficiency may simplify if the designs being compared have some characteristics in common. Section 3.1 compares basic staircase designs to their encompassing stepped wedge designs (such as the designs in Figure 1(b) and (a)): the designs share the same cluster-period size, *m*, and the number of clusters per sequence, thus the relative efficiency depends only on ψ and *S*. Section 3.2 compares extended basic staircase designs with more clusters per sequence to stepped wedge designs (such as the designs in Figure 1(c) and (a)). In Sections 3.2.1 and 3.2.2, the number of clusters per sequence is inflated by factors of (S+1)/2 and 2, respectively, thus the relative efficiency once again depends only on ψ and *S*. Section 3.3 compares basic staircase designs with larger cluster-period sizes to stepped wedge designs (such as the designs in Figure 1(d) and (a)): only the number of clusters per sequence cancels in the relative efficiency expression, but *a* and ψ depend on the cluster-period size and therefore differ between designs.

## 3 Relative design efficiency

### 3.1 Embedded staircase design versus encompassing stepped wedge design

**Figure 3.**
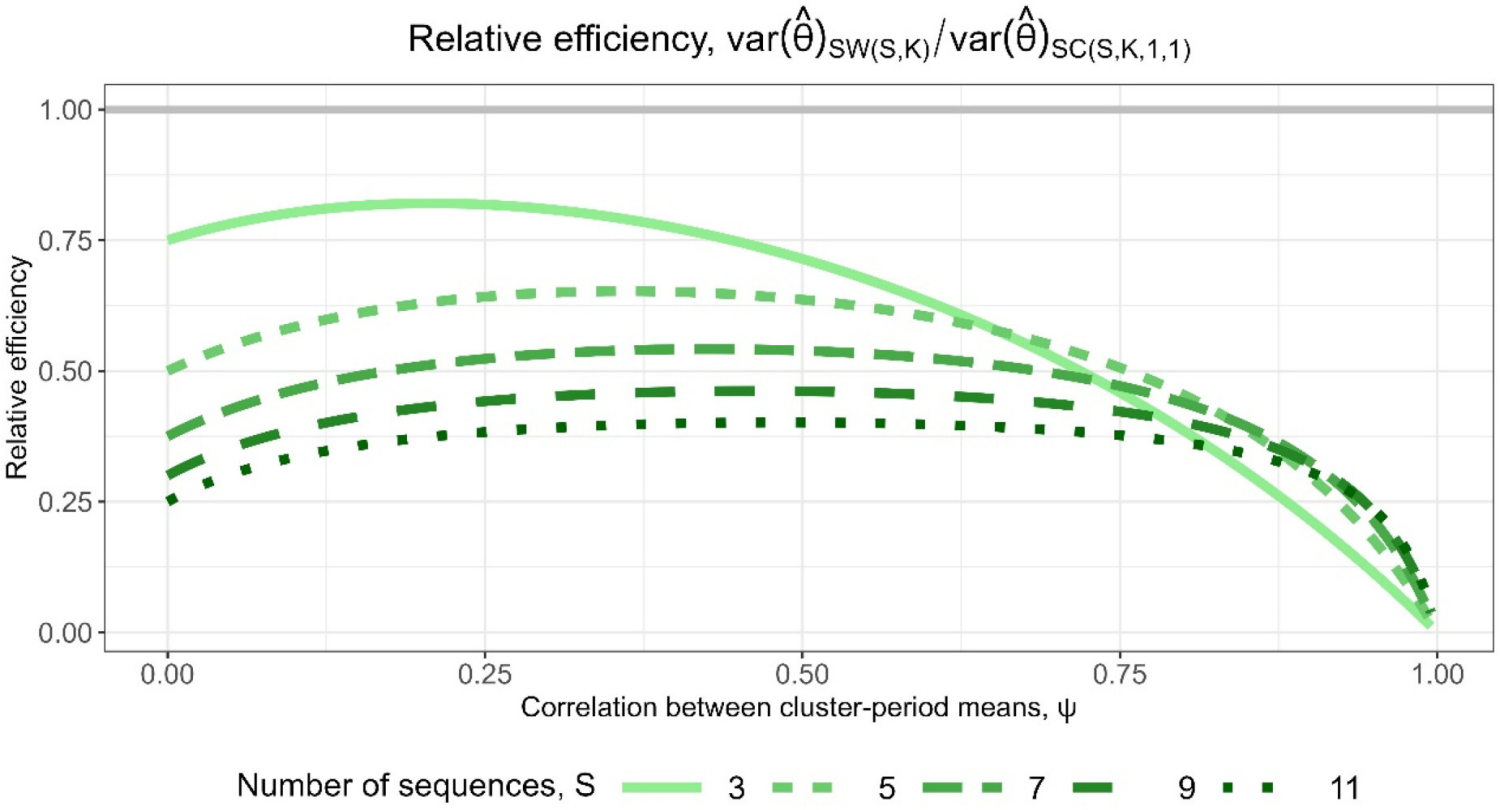
Relative efficiency for SC(S,K,1,1) embedded basic staircase designs compared to SW(S,K) stepped wedge designs, with S=3,5,7,9 and 11 sequences, for the block-exchangeable intracluster correlation structure assuming categorical period effects.

**Figure A1.**
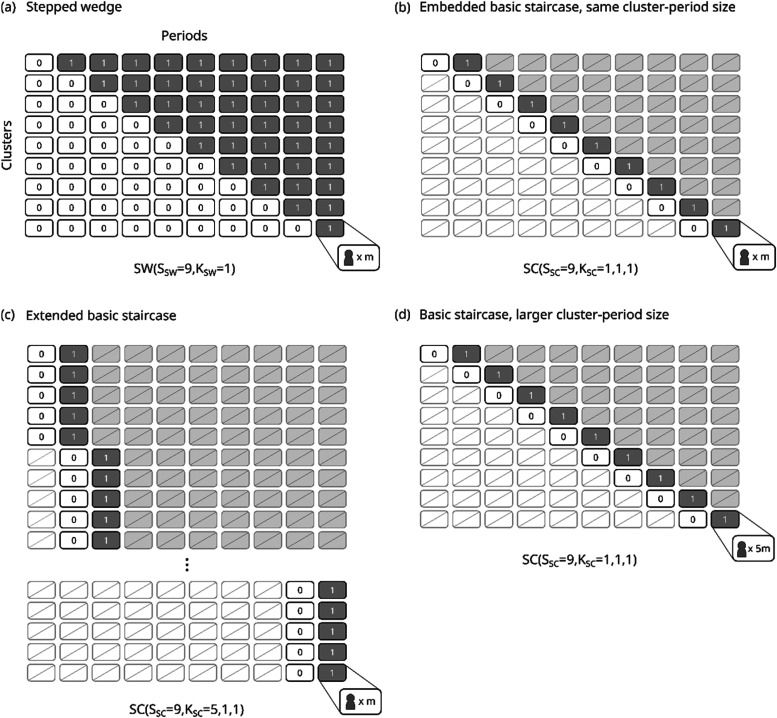
Design schematics for the nine-sequence designs to be compared, shown with a fixed number of clusters per sequence for illustrative purposes: (a) a stepped wedge design with one cluster per sequence and a cluster-period size of m , (b) a basic staircase design with one cluster per sequence and a cluster-period size of m , (c) an extended basic staircase design with five clusters per sequence and a cluster-period size of m , and (d) a basic staircase design with one cluster per sequence and a larger cluster-period size of 5m .

We will first compare the efficiency of a standard stepped wedge design to its embedded basic staircase design (Figure 1(b) depicts an example with three sequences and one cluster per sequence and Figure A1(b) depicts an example with nine sequences and one cluster per sequence). All designs have the same cluster-period size, *m*, and *K* clusters allocated to each sequence. Note that since the designs also have the same number of sequences $\left(S_{SC}=S_{SW}=S\right)$ and we are assuming a repeated cross-sectional sampling scheme, the embedded basic staircase design requires fewer participants than the encompassing stepped wedge design: 2SKm compared to S(S+1)Km participants. Figure 3 displays a line plot of the relative efficiencies for designs with S=3,5,7,9 and 11 sequences against ψ , the correlation between cluster-period means from equation (3). As expected, the relative efficiencies are all <1: the embedded basic staircase designs have fewer total participants than their encompassing stepped wedge designs, and are therefore less precise. The relative efficiencies are closest to 1 for small to moderate values of ψ and decrease down to zero as ψ increases up to 1. Designs with fewer sequences tend to have higher relative efficiencies, but as ψ approaches 1, the relative efficiencies for designs with different numbers of sequences are all similarly low. Notably, the relative efficiencies in most cases are out of proportion with the reduced sample size requirements of the staircase designs: embedded staircase designs with S=3,5,7,9 and 11 sequences require approximately 50%,33.3%,25%,20% and 16.7% of the participants required by the corresponding stepped wedge designs, yet the relative efficiencies are typically greater than these levels except when ψ is near one. We discuss the rationale for the patterns observed in Figure 3 in Section 5.

**Figure 4.**
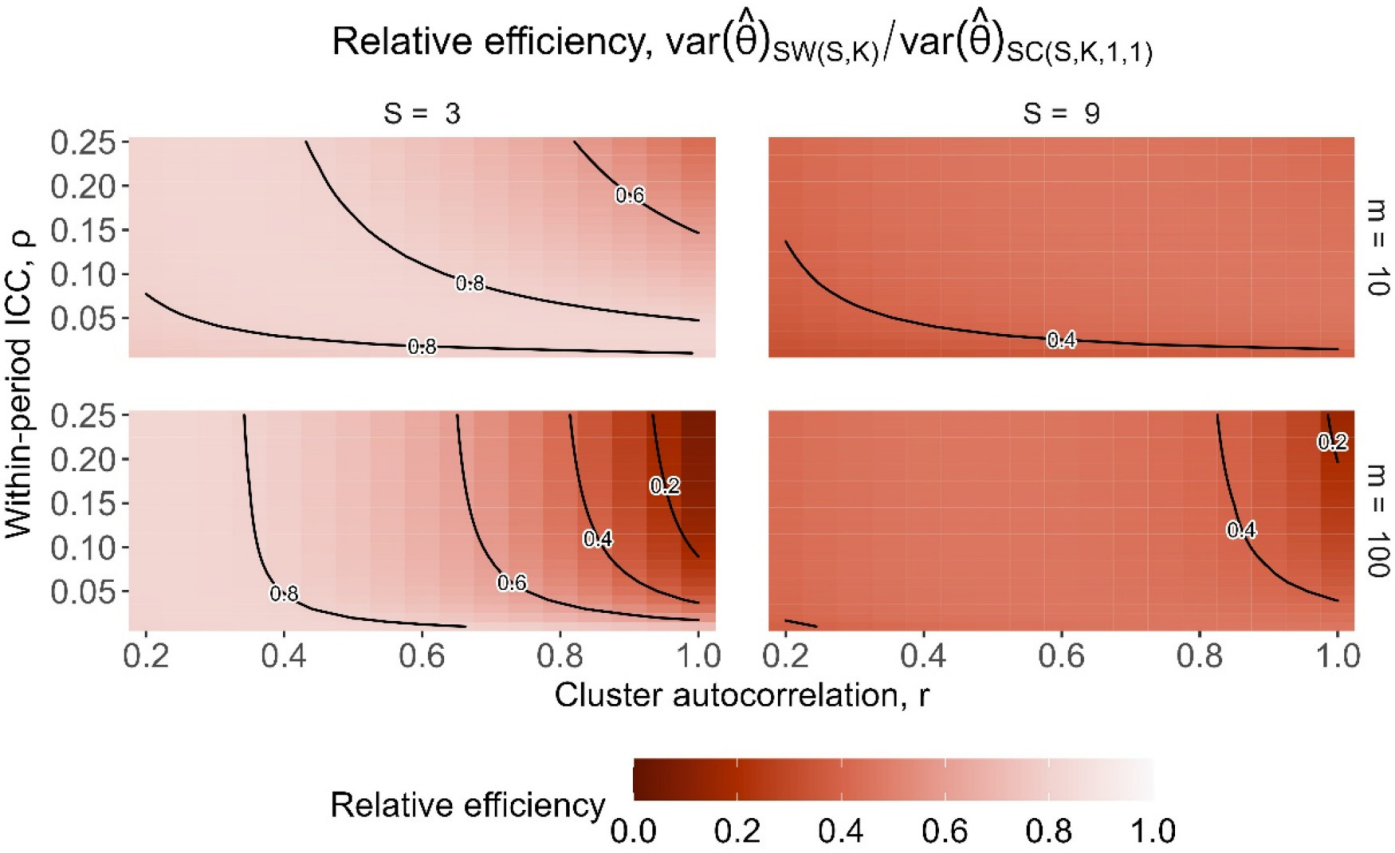
Relative efficiency for SC(S,K,1,1) embedded basic staircase designs compared to SW(S,K) stepped wedge designs, with S=3 sequences (left column) and S=9 sequences (right column), and with cluster-period sizes of m=10 (top row) and m=100 (bottom row), for the block-exchangeable intracluster correlation structure assuming categorical period effects.

To put the results in a more direct trial context, we also show the relative efficiencies as contour plots for different values of the underlying parameters of ψ : the cluster-period size and correlation parameters. Figure 4 displays the relative efficiencies for designs with m=10 participants per cluster-period (top row) and m=100 participants per cluster-period (bottom row), for designs with three sequences (left column) and nine sequences (right column). Each of the four contour plots shows the relative efficiencies for a range of correlation values: the within-period ICC, ρ , along the *y*-axis ranging from 0.01 to 0.25, and the cluster autocorrelation, *r*, ranging from 0.2 to 1 along the *x*-axis. As expected, the patterns of relative efficiency in Figure 4 are similar to the pattern of values for ψ shown in Figure 2, but with both very low and high values of ψ corresponding to low relative efficiency. Lower relative efficiencies mainly arise for trial settings with large values of both ρ and *r* (where *r* is more influential than ρ ) which correspond to large values of ψ . The relative efficiencies are also generally lower for designs with a larger cluster-period size of 100 compared to 10 together with large values of r : larger values of *m* cause ψ to approach the value of *r*, and so as *r* approaches one, so too does ψ . Relative efficiencies tend to be higher (i.e. closer to one) for smaller values of *r* between around 0.2 and 0.8 which correspond to moderate values of ψ . For the nine-sequence designs compared to the three-sequence designs, the relative efficiencies are generally lower and have less variation in magnitude, as was also shown in Figure 3.

### 3.2 Extended basic staircase design versus stepped wedge design

#### 3.2.1 The same total number of participants as the stepped wedge design

**Figure 5.**
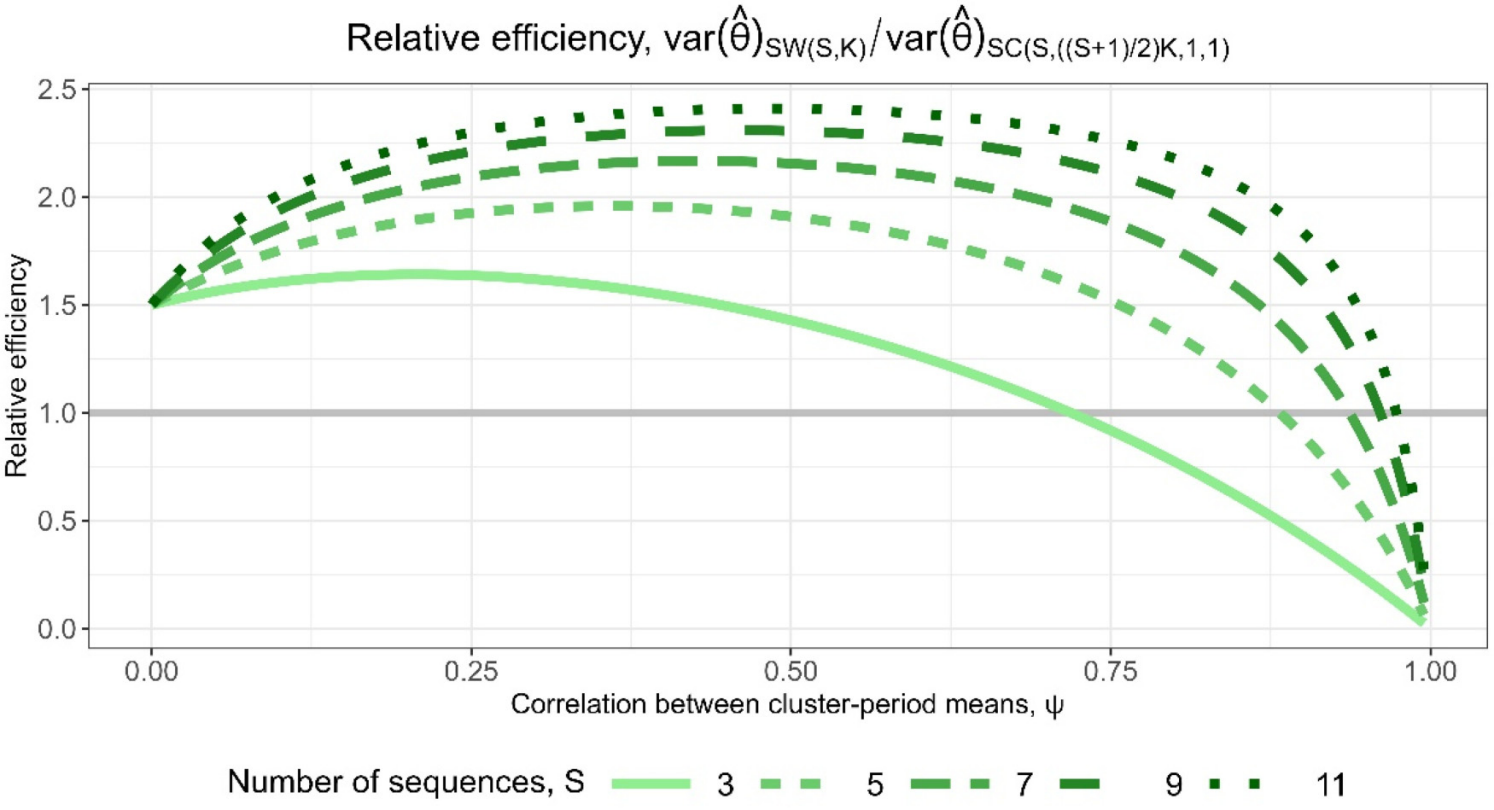
Relative efficiency for SC(S,qK,1,1) extended staircase designs compared to SW(S,K) stepped wedge designs, with S=3,5,7,9 and 11 sequences and q=(S+1)/2=2,3,4,5 and 6 , respectively, for the block-exchangeable intracluster correlation structure assuming categorical period effects.

We now compare stepped wedge and basic staircase designs with the same total number of participants. These designs have the same number of sequences, the same cluster-period size, but the stepped wedge designs have just $K_{SW}=K$ clusters per sequence while the basic staircase designs have $K_{SC}=[(S+1)/2]K$ clusters allocated to each sequence (such as in Figure 1(c) for a three-sequence design, shown with two clusters per sequence, and in Figure A1(c) for a nine-sequence design, shown with five clusters per sequence). We will refer to these basic staircase designs with additional clusters as ‘extended staircase designs’. Figure 5 displays the relative efficiencies against ψ for designs with different numbers of sequences. Note that, compared to the relative efficiencies shown in Figure 3, the relative efficiencies in Figure 5 for each value of *S* are inflated by a factor of (S+1)/2 . The extended staircase designs are typically more precise than the stepped wedge designs, except for large values of ψ , where all relative efficiencies decrease toward zero. The ordering of the lines is now reversed compared to Figure 3: designs with a greater number of sequences have higher relative efficiencies. For three-sequence designs, the relative efficiencies are as high as 1.6, while for 11-sequence designs, the relative efficiencies are as high as 2.4.

**Figure 6.**
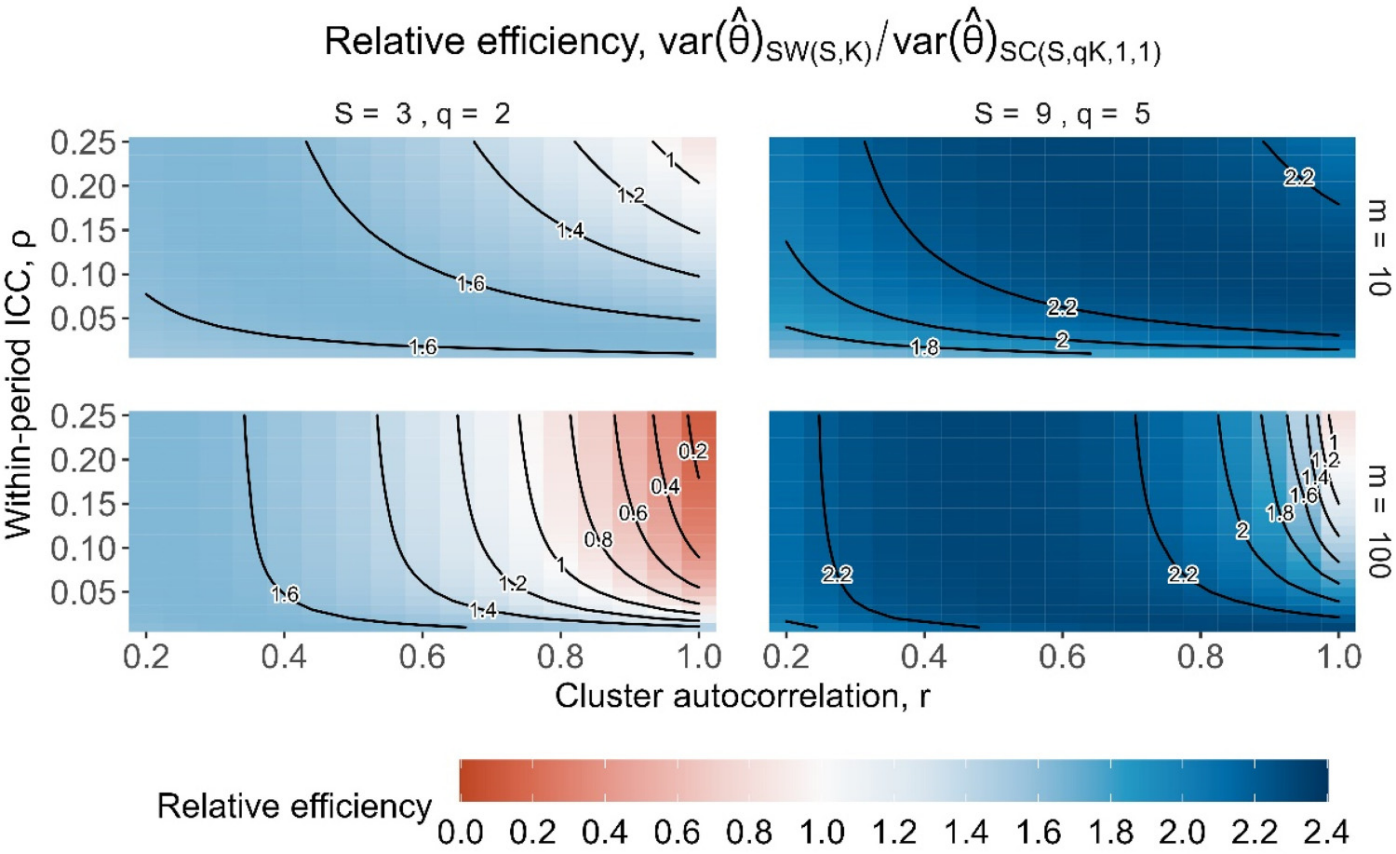
Relative efficiency for SC(S,qK,1,1) extended staircase designs compared to SW(S,K) stepped wedge designs, with S=3 sequences and q=2 (left column) and S=9 sequences and q=5 (right column), and with cluster-period sizes of m=10 (top row) and m=100 (bottom row), for the block-exchangeable intracluster correlation structure assuming categorical period effects.

Figure 6 shows the relative efficiencies for designs with three sequences (left column) and nine sequences (right column), for cluster-period sizes of 10 (top row) and 100 (bottom row), assuming categorical period effects. Since the relative efficiencies involving extended staircase designs are inflated by a factor of (S+1)/2 compared to those involving embedded staircase designs, the patterns of relative efficiencies are similar to those in Figure 3 but higher and covering a wider range of values. Aside from the settings with large ρ and *r*, where ψ is near one, the extended staircase design is as or more precise than the stepped wedge design for the same total number of participants.

#### 3.2.2 Fewer total participants than the stepped wedge design

The extended staircase designs in Section 3.2.1 with (S+1)/2 times as many clusters were typically more precise than the stepped wedge designs for the same total number of participants. However, for large *S* this inflation factor becomes large, while the number of available clusters is often limited. Here we consider extended staircase designs with a more modest inflation factor of 2, that is, twice as many clusters, to see whether the staircase designs can be as or more precise than stepped wedge designs for fewer total participants. Figure S1 in Section B.1 of the Supplemental Material displays the relative efficiencies against ψ for designs with 3, 5, 7, 9 and 11 sequences. The three-sequence extended staircase design has the same total number of participants as the stepped wedge as shown in Figure 5 while the other extended staircase designs have increasingly fewer total participants relative to the corresponding stepped wedge designs as the number of sequences increases. With the exception of large values of ψ for which all of the relative efficiencies are still near zero, the extended staircase designs with three and five sequences are typically more precise, the seven-sequence staircase design is around as precise, and the nine- and 11-sequence designs are slightly less precise than the corresponding stepped wedge designs. Figure S2 in the Supplemental Material displays the corresponding contour plots for five- and nine-sequence staircase designs, each with twice as many clusters per sequence as the stepped wedge designs (results for the three-sequence designs are already displayed in Figure 6). The five-sequence staircase designs are as or more precise than the stepped wedge designs, with relative efficiencies ranging from 1 up to 1.4, for designs with a small cluster-period size of 10 (top left), and for a large cluster-period size of 100 (bottom left) for cluster autocorrelation values less than around 0.8. For the nine-sequence designs, the staircase designs are slightly less precise than the stepped wedge designs, with relative efficiencies of around 0.8–0.9 across the majority of the plots.

### 3.3 Basic staircase design with larger cluster-period size versus stepped wedge design

#### 3.3.1 The same total number of participants as the stepped wedge design

Here we compare stepped wedge and basic staircase designs that again have the same total number of participants, where the cluster-period size for the basic staircase designs is larger than that for the stepped wedge designs and the designs have the same number of sequences and *K* clusters per sequence (Figure 1(d) depicts an example with three sequences and one cluster per sequence and Figure A1(d) depicts an example with nine sequences and one cluster per sequence). Letting $m_{SC}$ and $m_{SW}$ denote the cluster-period sizes of the staircase and stepped wedge designs, respectively, the designs have the same total number of participants when $m_{SC}=[(S+1)/2]m_{SW}$ . As the number of sequences increases, staircase designs require increasingly larger cluster-period sizes relative to the stepped wedge design to possess the same total number of participants: for three-sequence designs, $m_{SC}/m_{SW}=2$ (i.e. the number of participants in each cluster-period in the staircase design would be double that of the stepped wedge design), and for nine-sequence designs, $m_{SC}/m_{SW}=5$ . Note that for these comparisons, the relative efficiency cannot be expressed as a function of only *S* and ψ as previously, as ψ and *a* are functions of the cluster-period size and therefore differ between the staircase and stepped wedge designs.

**Figure 7.**
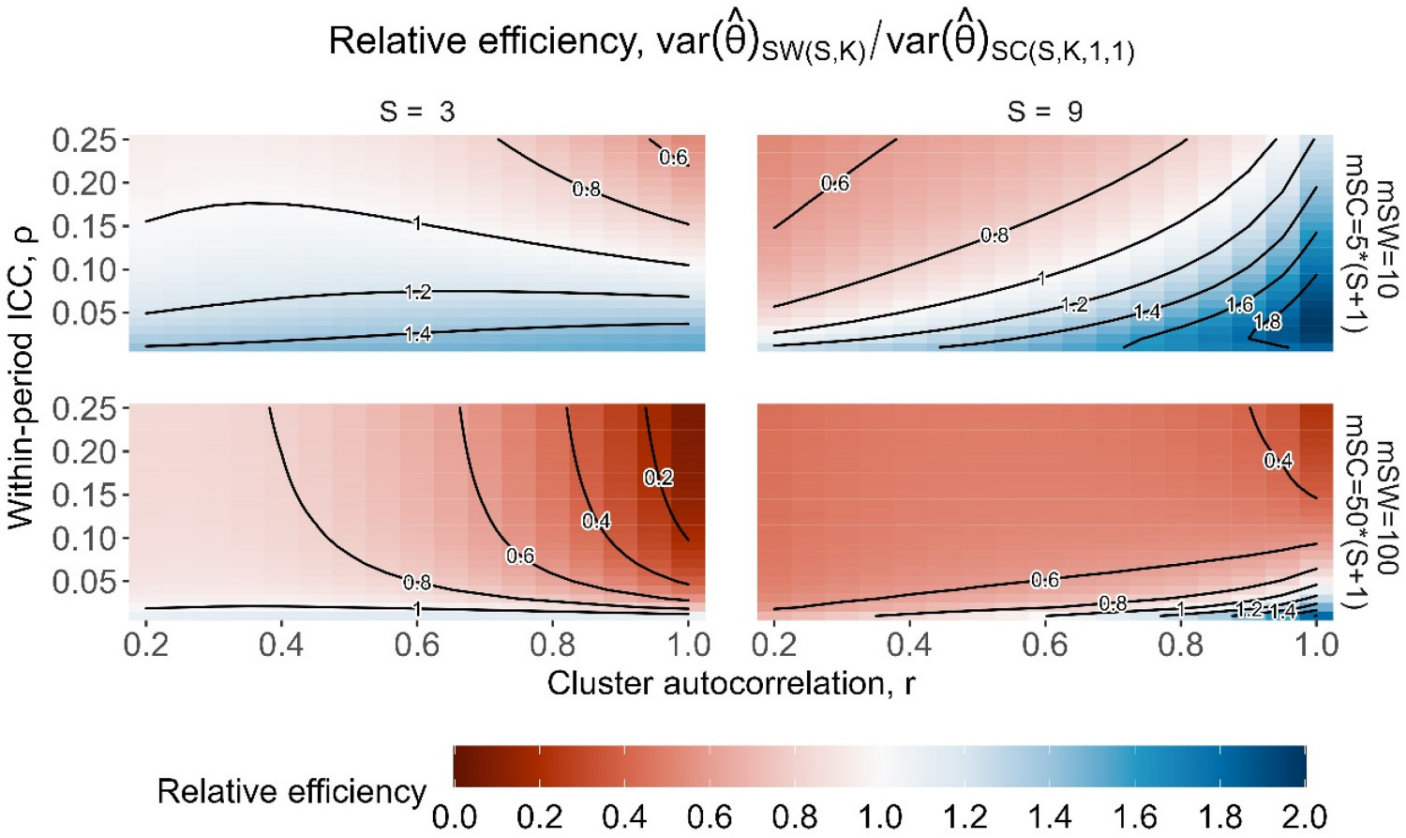
Relative efficiency for SC(S,K,1,1) basic staircase designs compared to SW(S,K) stepped wedge designs, with S=3 sequences (left column) and S=9 sequences (right column), and with cluster-period sizes of $m_{SW}=10$ and $m_{SC}=20$ (top left), $m_{SW}=10$ and $m_{SC}=50$ (top right), $m_{SW}=100$ and $m_{SC}=200$ (bottom left) and $m_{SW}=10$ and $m_{SC}=500$ (bottom right), for the block-exchangeable intracluster correlation structure assuming categorical period effects.

Figure 7 displays the relative efficiencies for three-sequence designs (left column) and nine-sequence designs (right column), for cluster-period sizes of $m_{SW}=10$ and $m_{SC}=2m_{SW}=20$ (top left), $m_{SW}=10$ and $m_{SC}=5m_{SW}=50$ (top right), $m_{SW}=100$ and $m_{SC}=2m_{SW}=200$ (bottom left), and $m_{SW}=100$ and $m_{SC}=5m_{SW}=500$ (bottom right). For the three-sequence designs (left column), there are broad regions with a relative efficiency close to 1, more so for a smaller cluster-period size, suggesting that the two different designs would yield similar statistical power in these settings. The relative efficiencies are the lowest for large ρ and *r* values (i.e. large values of ψ for both designs), as low as 0.53 for the designs where $m_{SW}=10$ (top row) and as low as 0.07 for the designs where $m_{SW}=100$ (bottom row). However, along the bottom of each of the subplots, for very small ρ values, relative efficiencies are generally above 1, as high as 1.53 for the designs where $m_{SW}=10$ , and up to 1.14 for designs where $m_{SW}=100$ . For the nine-sequence designs (right column), the patterns of relative efficiency are slightly different to those for the three-sequence designs. Settings with large ρ and small *r*, as well as large ρ , *r*, and $m_{SW}$ yield some of the lowest relative efficiencies, as low as 0.22, and settings with small ρ and large *r* yield the highest relative efficiencies, up to 1.98.

#### 3.3.2 Fewer total participants than the stepped wedge design

Here, rather than inflating the cluster-period size of the basic staircase designs by a factor of (S+1)/2 , we compare basic staircase designs that have cluster-period sizes that are 25% and 50% larger than those of the stepped wedge designs. Figure S3 in Section B.2 of the Supplemental Material displays the relative efficiencies for staircase designs with a 25% increase in their cluster-period size compared to the stepped wedge designs, where the staircase designs have 62.5% and 25% of the total number of participants as the stepped wedge, for three- and nine-sequence designs. While the staircase designs are typically less precise than the stepped wedge designs, the three-sequence designs with cluster-period sizes of $m_{SW}=10$ and $m_{SC}=12.5$ (top left) have similar precision for within-period ICC values less than around 0.1, across all cluster autocorrelation values ranging from 0.2 to 1. Design comparisons for staircase designs with a 50% increase in cluster-period size are shown in Figure S4 of the Supplemental Material, where the three- and nine-sequence staircase designs have 75% and 30% of the total number of participants as the stepped wedge designs, respectively. The relative efficiencies are all slightly higher than in Figure S3 of the Supplemental Material, as expected. For the three-sequence designs with cluster-period sizes of $m_{SW}=10$ and $m_{SC}=15$ (top left), the relative efficiencies are between 1.0 and 1.2 for within-period ICC values less than around 0.1, indicating that this staircase design with fewer total participants is as or more precise than the stepped wedge design.

## 4 Reimagining a stepped wedge design as a staircase design: The PROMPT trial

**Figure 8.**
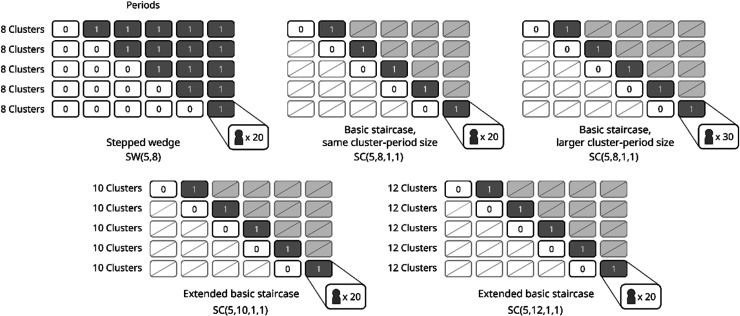
Design schematics for the designs inspired by the PROMPT trial: a stepped wedge design (top left), a basic staircase design with the same cluster-period size as the stepped wedge design (top middle), a basic staircase design with a 50% larger cluster-period size than the stepped wedge design (top right), each with five sequences $\left(S_{SW}=S_{SC}=5\right)$ and eight clusters per sequence $\left(K_{SW}=K_{SC}=8\right)$ and spanning six periods, and five-sequence extended basic staircase designs with 10 clusters per sequence (bottom left) and 12 clusters per sequence (bottom right).

In this section, we consider a stepped wedge trial inspired by the PROMPT trial^23^ which was a stepped wedge trial aiming to test whether a psychosocial intervention rolled out across different cancer treatment centres (the clusters) could reduce cancer patients’ depression scores. While this trial included just five clusters, we will instead consider a stepped wedge design with five sequences spanning six periods, with eight clusters randomly assigned to each sequence, for a total of 40 clusters (Figure 8, top left). An exchangeable correlation structure with an ICC of ρ=0.03 was assumed in the PROMPT trial, but here we will assume a block-exchangeable correlation structure as considered in Section 3. For the stepped wedge design we consider with a cluster-period size of 20 as in the PROMPT trial, an ICC of 0.03 corresponding to a design with six periods under exchangeable correlation aligns with, among other possible combinations, a within-period ICC of ρ=0.032 and a cluster autocorrelation of r=0.93 under block-exchangeable correlation^24^; the correlation between cluster-period means for this trial setting is ψ=0.37 . This stepped wedge design would have 87.3% power to detect a standardised effect size of 0.15 at the two-sided 5% significance level.

Now suppose that the basic staircase design embedded in the stepped wedge design were to be implemented instead (Figure 8, top middle). This design has 80 cluster-periods of measurement compared to 240 in the stepped wedge design. With the same cluster-period size of 20 and assuming categorical period effects, this basic staircase design would have 70.7% power to detect an effect size of 0.15 with a two-sided significance level of 5%. The relative efficiency of this embedded staircase design compared to the stepped wedge design is 0.653 (as can also be seen for the five-sequence designs in Figure 3 when ψ=0.37 ), and so the estimator corresponding to the embedded staircase design with no modification is 34.7% less precise than that from the stepped wedge design. However, we can drastically increase its relative efficiency if it were possible to recruit more participants in each cluster or if additional clusters were available.

A basic staircase design with a larger cluster-period size of 30 (as depicted in Figure 8, top right), that is, a 50% increase, would have 84.1% power and a relative efficiency of 0.91 (only 9% less precise) compared to the stepped wedge design with a cluster-period size of 20. Notably, this design would require 50% fewer total participants, with only 2400 total participants required across the 80 cluster-periods rather than 4800 total participants across the 240 cluster-periods in the stepped wedge design. Figure S1 of the Supplemental Material shows that a five-sequence extended basic staircase design with twice as many clusters as the stepped wedge would yield a relative efficiency of around 1.25 when ψ=0.37 . However, a doubling of the number of cancer centres that are included in the trial from 40 to 80 may not be possible. A basic staircase design with the same cluster-period size of 20 but with 50 clusters (a 25% increase) where 10 clusters are randomised to each sequence (Figure 8, bottom left) would have 79.9% power and a relative efficiency of 0.816 compared to the stepped wedge design. If more clusters were available, then a basic staircase with 60 clusters (a 50% increase) where 12 clusters are randomised to each sequence (Figure 8, bottom right) would have 86.6% power and a relative efficiency of 0.979 compared to the stepped wedge design. These extended staircase designs would require 2000 and 2400 participants, respectively (rather than the 4800 in the stepped wedge design).

We note that these power and relative efficiency calculations use formulae based on asymptotic properties. For designs with a small number of clusters, theoretical power may not reflect actual power; however, for the designs considered here with 40–60 clusters, the empirical power and relative efficiency values align fairly closely with the theoretical values (see Section C of the Supplemental Material for details).

## 5 Discussion

The basic staircase design is a particularly lean and potentially powerful alternative to the stepped wedge design. Basic staircase designs make use of only the immediate pre- and post-switch periods for each cluster, which are the cluster-periods in a stepped wedge design that have been shown to contribute a great deal of information to the estimation of the treatment effect.^4^ At the trial design stage, trialists who are considering conducting a stepped wedge design may wish to also consider a basic staircase design if the candidate stepped wedge design yields power greater than what is required, or if there is some flexibility in the number of participants that could be measured in each cluster-period or in the number of available clusters. Without modification to cluster-period size or the number of participating clusters, the embedded staircase design contains a subset of the participants in the encompassing stepped wedge design and so will always be less powerful to some degree. However, the loss of efficiency associated with the use of an embedded staircase design instead of the stepped wedge design is far less than the proportionate reduction in the number of participants for most realistic trial settings. Moreover, in many realistic settings, that is, for many realistic combinations of correlation parameter values, the embedded staircase design is only slightly less efficient than the stepped wedge design. If the cluster-period size or number of clusters could feasibly be higher, then a basic staircase design can be more powerful than the stepped wedge design for the same number of total participants, as shown in Sections 3.2.1 and 3.3.1. Some basic staircase designs can even achieve power equal to or greater than stepped wedge designs while requiring measurements on fewer total participants, as was seen in Sections 3.2.2 and 3.3.2. Thus, depending on the design itself, the assumed correlation structure and parameters, and the desired effect size, there will be scenarios in which the staircase design offers sufficient power, while placing less of a burden on participating clusters than would the comparator stepped wedge design.

The variation in the relative efficiencies for the design comparisons in Section 3.1 can in part be explained by understanding when the treatment effect estimators for the stepped wedge and staircase designs do and do not have similar forms. Matthews and Forbes^25^ showed that the stepped wedge design estimator uses a weighted combination of ‘vertical’ (within-column) and ‘horizontal’ (row-column) estimators of the treatment effect. In particular, the vertical estimator is a weighted combination of an intuitive form of within-column contrasts, namely the mean of the outcomes of the intervention cells minus the mean of the outcomes of the control cells within each column (period). Grantham et al.^8^ showed that the basic staircase design estimator where categorical time effects are assumed is also a vertical estimator based on this same form of contrasts, simplified to one intervention and one control cell within each column. Note that the key results in Matthews and Forbes depend on the correlation between cluster-period means, ψ , which they define under an exchangeable correlation structure, but their results also apply to the block-exchangeable structure we considered through the definition of ψ as in equation (3). When ψ is large, the stepped wedge design estimator predominantly involves the horizontal estimator, and conversely when ψ is small, the estimator is predominantly the vertical estimator.^25^ Because the basic staircase design estimator resembles the form of the stepped wedge design vertical estimator, the variance of the basic staircase design estimator is generally most similar to that of the encompassing stepped wedge design estimator when the stepped wedge design estimator is dominated by the vertical estimator, which occurs when ψ is small. Conversely, the variances are most dissimilar when the stepped wedge design estimator is dominated by the horizontal estimator (large ψ ). These accord with the changes in relative efficiency under small and large values of ψ observed in Figure 3, specifically when the relative efficiency is closer to one for smaller values of ψ and tends to zero as ψ approaches one. Stepped wedge designs with a greater number of sequences (and hence a larger number of time periods) also give increased weight to the horizontal estimator as long as the correlation parameters are nonzero, and so these are also circumstances where the relative efficiency tends to be lower.

Across the comparisons between various basic staircase designs and a stepped wedge design, we observed that the relative efficiency is quite sensitive to the number of sequences and the strength of correlation between cluster-period means, ψ , and therefore the correlation parameter values and cluster-period size. These values will be context-specific and so a staircase design will be an efficient alternative to a stepped wedge design in some settings but not in others. For the PROMPT trial described in Section 4, for example, a basic staircase design would have been particularly appealing: with an increase to the cluster-period size from 20 to 30, a five-sequence basic staircase design with eight clusters assigned to each sequence would require 50% fewer participants to achieve similar power to a stepped wedge design. Alternatively, a five-sequence basic staircase design with a cluster-period size of 20 but with a 50% increase in the number of clusters would also require 50% fewer total participants than the stepped wedge design and yield similar power. For stepped wedge designs with a larger number of sequences, the embedded basic staircase design will retain a smaller proportion of the cluster-periods; more substantial rearrangement of measurements such as through additional clusters or larger cluster-period sizes would be required for a staircase design to maintain similar power to a stepped wedge design.

This article focused on situations where a block-exchangeable correlation structure and categorical period effects were assumed, for a repeated cross-sectional sampling scheme and designs without implementation periods. Other assumed forms for the ICC and time effects may be more appropriate for a given trial context, such as a discrete-time decay correlation structure and/or a linear effect of time over the trial periods. Section D of the Supplemental Material displays and describes relative efficiency results under these alternative assumptions. Results for a cohort sampling scheme and designs including implementation periods are provided in Section E of the Supplemental Material. In general, the relative efficiency results under these alternative assumptions, sampling schemes and design types are not vastly different from those in Section 3.

The relative efficiency results in this article apply to estimators obtained from linear mixed models for continuous outcomes and further work is required for other outcome types and/or the use of marginal models. Results for marginal models, for continuous and discrete outcomes, show that the cells near the time of the treatment switch in stepped wedge designs are typically the most information-rich cells.^7,26^ This is similar to results observed for the linear mixed model setting we explore, and suggests that embedded basic staircase designs comprised of only the cells before and after the treatment switch would be relatively efficient compared to the encompassing stepped wedge designs in these other settings too. However, the efficiency of staircase designs has not been directly compared to that of stepped wedge designs for binary and count outcomes using marginal models, and so the magnitude of the relative efficiencies may differ from our results.^7^ In contrast to the results obtained for the linear mixed model setting we consider, recent results for continuous outcomes modelled using marginal models under a working independence assumption show that some cells further from the treatment switch in the stepped wedge can have information content less than one. Thus, in this setting, embedded staircase designs can be even more efficient than the encompassing stepped wedge designs.^26^ This was shown for models with exchangeable and discrete-time decay correlation structures, but we would expect similar results for the block-exchangeable correlation structure primarily considered in this article.

This article considered when different basic staircase designs may be desirable over a stepped wedge design on the grounds of statistical efficiency, but feasibility and the associated trial costs would also factor into the choice of design. From a feasibility standpoint, a staircase design may hold greater appeal than a stepped wedge design in settings where data collection is particularly onerous. For example, in the INSPIRED stepped wedge trial assessing the impact of Palliative Care Needs Rounds in care homes,^3^ secondary outcomes of interest pertained to subjective measures of patients’ quality of death and dying that were captured through surveys conducted by care home staff. In that study, the researchers needed to reduce both the length and number of outcome measures to reduce the burden of data collection on the staff, and train a large number of staff per site in data collection due to high staff turnover. Had a staircase design been considered, clusters would have been involved for much less time and may have had greater short-term capacity to take a variety of measurements. In addition, perhaps fewer staff would need to be trained. In choosing among the staircase design variants, there are likely to be setting-specific constraints on, for instance, the number of clusters and the number of participants that could be measured in each cluster-period that may guide the choice. From a trial cost standpoint, the staircase design variants would have different associated costs relative to the cost of the stepped wedge design. For example, suppose a simple formula for the total trial cost accounts for the cost of including a cluster and the cost of including and measuring a participant such as that considered in Grantham et al.^27^ Then compared to the stepped wedge design, the embedded basic staircase would have a lower trial cost (an equal number of clusters but fewer total participants), the extended staircase would have a larger trial cost (greater number of clusters, same total participants), and the basic staircase with a larger cluster-period size would have the same trial cost (equal number of clusters and total participants). Additional factors could also affect the cost and feasibility; for example, it is possible that increasing the number of participants per cluster-period would require additional staff to take these measurements or implement the intervention. While beyond the scope of this article, it would be of interest to consider whether the additional cost and effort associated with including additional clusters or taking more measurements in each cluster-period would outweigh the benefits of increased precision that these staircase design variants would bring.

In conclusion, in this article, we have demonstrated that the basic staircase design is an alternative to the stepped wedge design that will, for a wide range of realistic design parameters, require fewer participants than the stepped wedge design but still have comparable power to detect treatment effects. Further, we have shown that there are scenarios in which slight increases in cluster-period sizes or the number of clusters recruited to a staircase design may lead to a design with greater power compared to the stepped wedge design, for the same total number of participants or even with fewer total participants. Whether such modifications would be feasible depends on the trial context: hence the appropriateness of the staircase design is heavily dependent on the setting in which a trial is being conducted. These observations are also dependent on design parameters, and thus we would encourage researchers who are planning to conduct a stepped wedge design to investigate staircase designs as potentially less burdensome alternatives.

## Supplemental Material

sj-pdf-1-smm-10.1177_09622802251317613 - Supplemental material for The relative efficiency of staircase and stepped wedge cluster randomised trial designs Supplemental material, sj-pdf-1-smm-10.1177_09622802251317613 for The relative efficiency of staircase and stepped wedge cluster randomised trial designs by Kelsey L Grantham, Andrew B Forbes, Richard Hooper and Jessica Kasza in Medical Research
