# Supplementary material for: The relative efficiency of staircase and stepped wedge cluster randomised trial designs
Source: Stat Methods Med Res. 2025 Feb 16;34(4):701–16. doi: 10.1177/09622802251317613 (PMC12075890; doi:10.1177/09622802251317613)
Supplement: sj-pdf-1-smm-10.1177_09622802251317613 - Supplemental material for The relative efficiency of staircase and stepped wedge cluster randomised trial designs [file sj-pdf-1-smm-10.1177_09622802251317613.pdf]

Supplemental Material

The relative efficiency of staircase and stepped wedge cluster randomised trial designs

Kelsey L. Grantham, Andrew B. Forbes, Richard Hooper, Jessica Kasza

A: Derivation of analytical variance of treatment effect estimator, stepped wedge design

A.1: Reduced variance expression

Suppose a complete stepped wedge design with S unique treatment sequences, each repeated K times. Then there are $S+1$ periods and the treatment sequences $\mathbf{X}_s$ are $(S+1)$-dimensional column vectors comprised of s zeros followed by $S+1-s$ ones.

If either categorical period or linear time period effects are assumed, then for the complete stepped wedge design, the variance of the treatment effect estimator reduces to (Grantham et al, 2020):

$$\text{var}(\hat{\theta})_{SW(S,K)} = \frac{1}{K}\left[\sum_{s=1}^{S}\mathbf{X}_s^\top \mathbf{V}_*^{-1}\mathbf{X}_s - \frac{1}{S}\left(\sum_{s=1}^{S}\mathbf{X}_s\right)^\top \mathbf{V}_*^{-1}\left(\sum_{s=1}^{S}\mathbf{X}_s\right)\right]^{-1}. \quad (1)$$

A.2: Variance expression with a block-exchangeable intracluster correlation structure

If we assume a block-exchangeable intracluster correlation structure, then the covariance matrix at the cluster-period mean level for a single cluster, assumed common across clusters, is an exchangeable matrix that can be written as

$$\mathbf{V}_* = \begin{pmatrix} a & b & \cdots & b \\ b & a & & \vdots \\ \vdots & & \ddots & b \\ b & \cdots & b & a \end{pmatrix} = (a-b)\mathbf{I} + b\mathbf{J}$$

where $\mathbf{I}$ is the $(S+1)\times(S+1)$ identity matrix and $\mathbf{J}$ is the $(S+1)\times(S+1)$ matrix of ones. The inverse of $\mathbf{V}_*$ can be written as (Li et al, 2019; Graybill, 1983):

$$\begin{aligned}\mathbf{V}_*^{-1} &= \frac{1}{a-b}\mathbf{I} - \frac{b}{(a-b)(a+Sb)}\mathbf{J} \\ &= \frac{1}{(a-b)(a+Sb)}\begin{pmatrix} a+(S-1)b & -b & \cdots & -b \\ -b & a+(S-1)b & & \vdots \\ \vdots & & \ddots & -b \\ -b & \cdots & -b & a+(S-1)b \end{pmatrix}.\end{aligned}$$

For simplicity going forward, let $g = \frac{a+(S-1)b}{(a-b)(a+Sb)}$ and $h = \frac{-b}{(a-b)(a+Sb)}$.

It can be shown that $\mathbf{X}_s^\top \mathbf{V}_*^{-1} \mathbf{X}_s = sg + s(s-1)h$ and so

$$\begin{aligned}\sum_{s=1}^{S} \mathbf{X}_s^\top \mathbf{V}_*^{-1} \mathbf{X}_s &= \sum_{s=1}^{S} [sg + s(s-1)h] \\ &= \left(\sum_{s=1}^{S} s\right) g + \left(\sum_{s=1}^{S} s^2 - \sum_{s=1}^{S} s\right) h \\ &= \frac{1}{2}S(S+1)g + \left[\frac{1}{6}S(S+1)(2S+1) - \frac{1}{2}S(S+1)\right] h \\ &= \frac{1}{2}S(S+1)g + \frac{1}{3}S(S^2-1)h.\end{aligned}$$

The other term in (1) can be written as

$$\begin{aligned}\left(\sum_{s=1}^{S} \mathbf{X}_s\right)^\top \mathbf{V}_*^{-1} \left(\sum_{s=1}^{S} \mathbf{X}_s\right) &= \begin{pmatrix}0 & 1 & 2 & \cdots & S\end{pmatrix} \mathbf{V}_*^{-1} \begin{pmatrix}0 & 1 & 2 & \cdots & S\end{pmatrix}^\top \\ &= \left(\sum_{s=1}^{S} s^2\right) g + \left(\sum_{s=1}^{S} s \sum_{s'=1}^{S} s' - \sum_{s=1}^{S} s^2\right) h \\ &= \frac{1}{6}S(S+1)(2S+1)g + \left(\frac{1}{2}S(S+1) \cdot \frac{1}{2}S(S+1) - \frac{1}{6}S(S+1)(2S+1)\right) h \\ &= \frac{1}{6}S(S+1)(2S+1)g + \frac{1}{12}S(S^2-1)(3S+2)h\end{aligned}$$

Therefore (1) can be simplified to

$$\begin{aligned}\mathrm{var}(\hat{\theta})_{SW(S,K)} &= \frac{1}{K}\left[\sum_{s=1}^{S} \mathbf{X}_s^\top \mathbf{V}_*^{-1} \mathbf{X}_s - \frac{1}{S}\left(\sum_{s=1}^{S} \mathbf{X}_s\right)^\top \mathbf{V}_*^{-1} \left(\sum_{s=1}^{S} \mathbf{X}_s\right)\right]^{-1} \\ &= \frac{1}{K}\left[\frac{1}{2}S(S+1)g + \frac{1}{3}S(S^2-1)h - \frac{1}{S}\left(\frac{1}{6}S(S+1)(2S+1)g + \frac{1}{12}S(S^2-1)(3S+2)h\right)\right]^{-1} \\ &= \frac{1}{K}\left[\left(\frac{1}{2}S(S+1) - \frac{1}{6}(S+1)(2S+1)\right) g + \left(\frac{1}{3}S(S^2-1) - \frac{1}{12}(S^2-1)(3S+2)\right) h\right]^{-1} \\ &= \frac{1}{K}\left[\frac{1}{12}(S^2-1)\left(2g + (S-2)h\right)\right]^{-1} \\ &= \frac{1}{K}\left\{\frac{1}{12}(S^2-1) \cdot \frac{2\left(a + (S-1)b\right) + (S-2)(-b)}{(a-b)(a+Sb)}\right\}^{-1} \\ &= \frac{12(a-b)(a+Sb)}{K(S^2-1)(2a+Sb)}.\end{aligned}$$

Note that for a simple exchangeable model where e.g. $a = \sigma_C^2 + \frac{\sigma_e^2}{m}$ and $b = \sigma_C^2$, this expression becomes

$$\mathrm{var}(\hat{\theta})_{SW(S,K)} = \frac{12\frac{\sigma_e^2}{m}\left[\frac{\sigma_e^2}{m} + (S+1)\sigma_C^2\right]}{K(S^2-1)\left[2\frac{\sigma_e^2}{m} + (S+2)\sigma_C^2\right]}$$

which matches (following some simple algebra) the analytical variance expression given in Hussey and Hughes (2007) for a stepped wedge design assuming exchangeable intracluster correlation and categorical time period effects.

B: Relative efficiency plots for staircase designs with fewer total participants than the stepped wedge

B.1: Extended basic staircase design with twice as many clusters

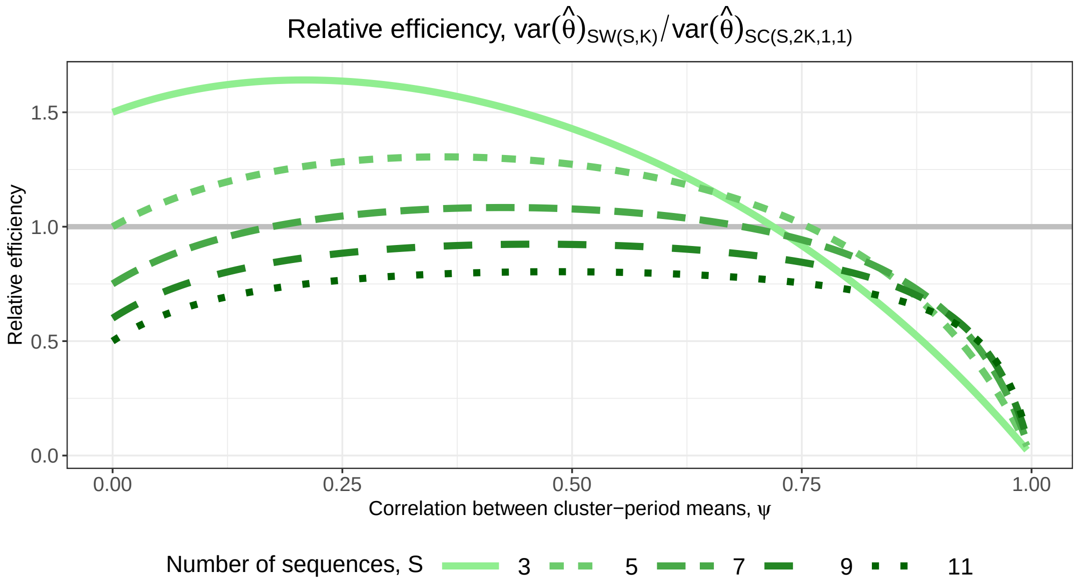

Figure S1: Relative efficiency for $SC(S, 2K, 1, 1)$ extended staircase designs compared to $SW(S, K)$ stepped wedge designs, with $S = 3, 5, 7, 9$ and 11 sequences, for the block-exchangeable intracluster correlation structure assuming categorical period effects.

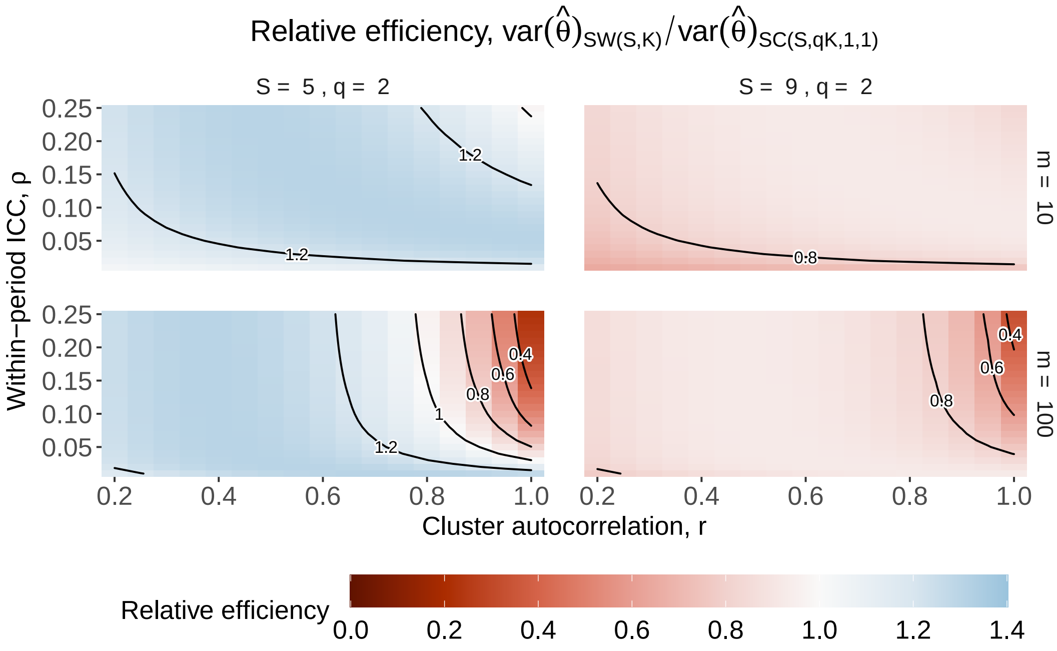

Figure S2: Relative efficiency for $SC(S, qK, 1, 1)$ extended staircase designs compared to $SW(S, K)$ stepped wedge designs, with $S = 5$ sequences and $q = 2$ (left column) and $S = 9$ sequences and $q = 2$ (right column), and with cluster-period sizes of $m = 10$ (top row) and $m = 100$ (bottom row), for the block-exchangeable intracluster correlation structure assuming categorical period effects.

B.2: Basic staircase design with 25% and 50% larger cluster-period sizes

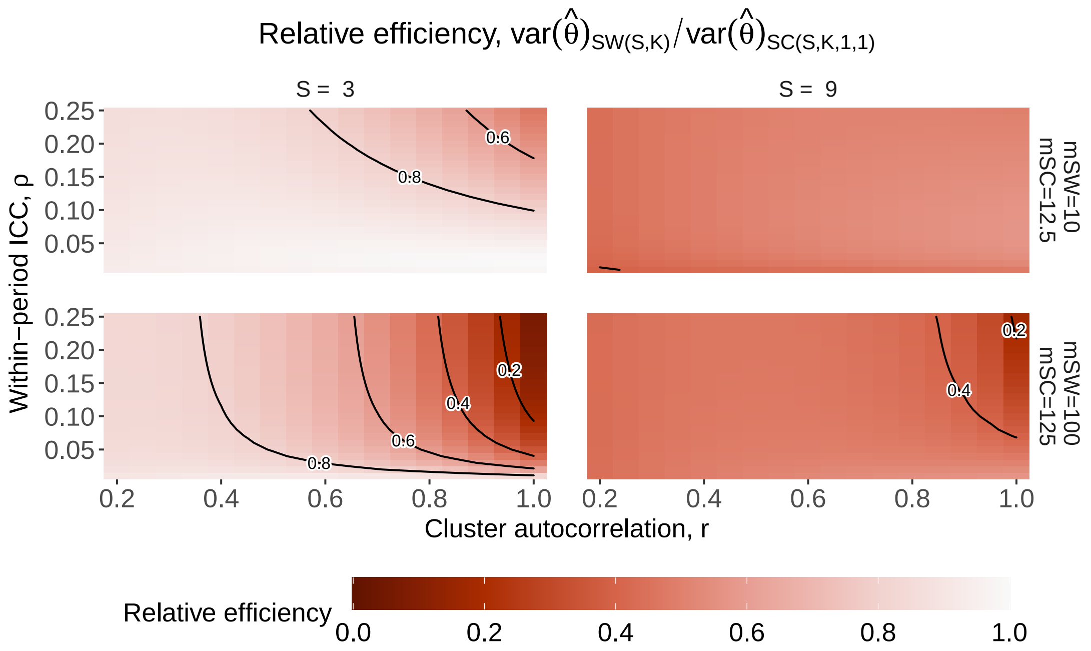

Figure S3: Relative efficiency for $SC(S, K, 1, 1)$ basic staircase designs compared to $SW(S, K)$ stepped wedge designs, with $S = 3$ sequences (left column) and $S = 9$ sequences (right column), and with cluster-period sizes of $m_{SW} = 10$ and $m_{SC} = 12.5$ (top) and $m_{SW} = 100$ and $m_{SC} = 125$ (bottom), for the block-exchangeable intracluster correlation structure assuming categorical period effects.

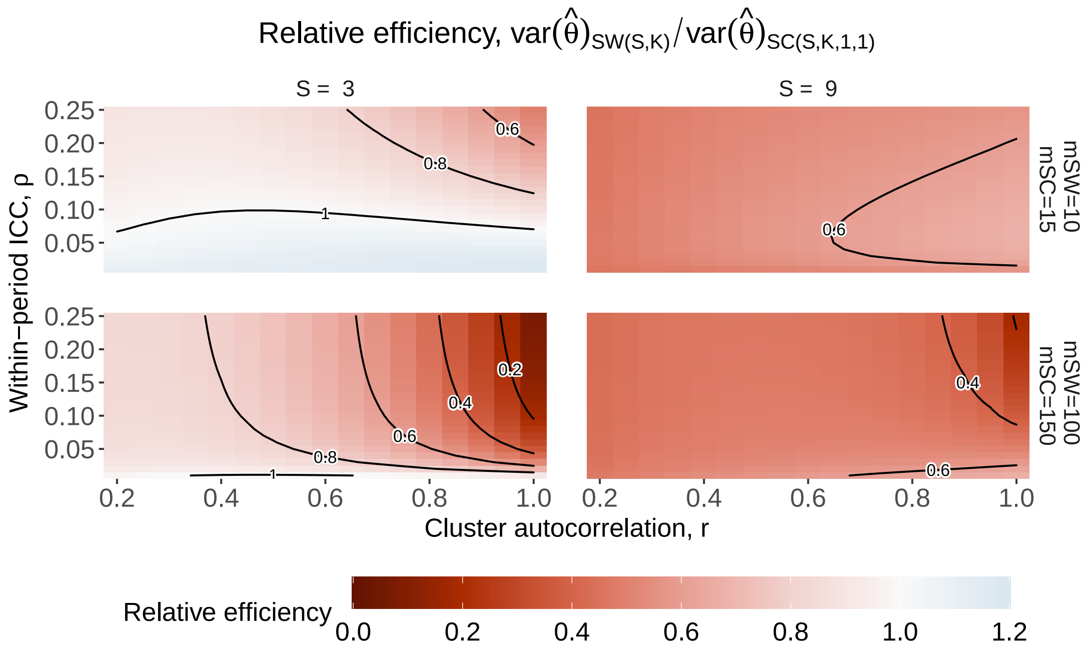

Figure S4: Relative efficiency for $SC(S, K, 1, 1)$ basic staircase designs compared to $SW(S, K)$ stepped wedge designs, with $S = 3$ sequences (left column) and $S = 9$ sequences (right column), and with cluster-period sizes of $m_{SW} = 10$ and $m_{SC} = 15$ (top) and $m_{SW} = 100$ and $m_{SC} = 150$ (bottom), for the block-exchangeable intracluster correlation structure assuming categorical period effects.

C: Simulation study to calculate empirical power and relative efficiency

Since the formulae used in Section 4 to calculate power and relative efficiency are based on asymptotic properties and may not reflect actual power, we also calculated empirical power and relative efficiency for the designs with a small simulation study. For each of the four trial configurations ($K = 8$ and $m = 20$, $K = 8$ and $m = 30$, $K = 10$ and $m = 20$, and $K = 12$ and $m = 20$), we simulated 1000 trial datasets for a 5-sequence stepped wedge trial with a block-exchangeable within-cluster correlation structure (with $\rho = 0.032$ and $r = 0.93$) and retained the relevant subset of measurements corresponding to the basic staircase design. We then fit a model assuming a block-exchangeable within-cluster correlation structure to the simulated trial data, and calculated empirical study power for the designs as the percentage of times the hypothesis test of a null treatment effect was rejected at the two-sided 5% significance level. We also calculated the empirical relative efficiency for each of the staircase designs compared to the stepped wedge design ($S = 5$, $K = 8$, $m = 20$) by taking the ratio of the empirical variance for the stepped wedge design to the empirical variance of the particular staircase design. Finally, we calculated the empirical type I error rate to determine whether it was close to the nominal 5% level. To do this, we defined a second set of simulated trial datasets similar to the first set but with a null treatment effect, and calculated the percentage of times the hypothesis test of a null treatment effect was incorrectly rejected. Code to replicate our simulation is available at https://github.com/klgrantham/compare-SC-SW.

Table 1 summarises the results, showing theoretical and empirical power along with the theoretical and empirical relative efficiencies and empirical type I error rates. The empirical type I error rates for each of the simulations are all close to the nominal 5% level (within two Monte Carlo standard errors (MCSE) (1.4%) of the nominal level for all designs), most being slightly lower. Empirical power and relative efficiency values align fairly closely with the theoretical values for all designs: empirical power estimates are within two MCSE of the theoretical values for all designs except the basic staircase design with $S = 5$, $K = 10$, and $m = 20$, which is within 2.5 MCSE. The empirical relative efficiencies of the staircase designs compared to the stepped wedge design are slightly lower than the theoretical relative efficiencies for two of the four staircase designs, and slightly higher for the other two designs.

**Design**	**Theoretical power**	**Empirical power**	**Theoretical relative efficiency**	**Empirical relative efficiency**	**Empirical type I error rate**
**Stepped wedge**					
$S = 5$, $K = 8$, $m = 20$	87.3%	86.1%	-	-	5.3%
**Basic staircase**					
$S = 5$, $K = 8$, $m = 20$	70.7%	68.9%	0.653	0.683	3.9%
$S = 5$, $K = 8$, $m = 30$	84.1%	81.9%	0.910	0.854	4.8%
$S = 5$, $K = 10$, $m = 20$	79.9%	76.7%	0.816	0.763	4.2%
$S = 5$, $K = 12$, $m = 20$	86.6%	86.5%	0.979	0.993	3.7%

Table 1: Empirical power, relative efficiency, and type I error rate compared to theoretical values for a stepped wedge design and several staircase designs. Trial data was generated with a block-exchangeable within-cluster correlation structure with $\rho = 0.032$ and $r = 0.93$.

D: Relative efficiency plots under alternative modelling assumptions

D.1: Assuming discrete-time decay correlation

The broad patterns of relative efficiencies under the discrete-time decay correlation structure are generally similar to those under the block-exchangeable structure, but with more extreme relative efficiencies over the ranges of parameter values (Figures S5-S7). We note that we cannot directly compare results across correlation structures for the same correlation parameters as these models are parameterised differently. Since we are considering a basic staircase design, for which the block-exchangeable and discrete-time decay structures are equivalent, only the treatment effect estimator variance for the stepped wedge design is impacted by assuming discrete-time decay correlation.

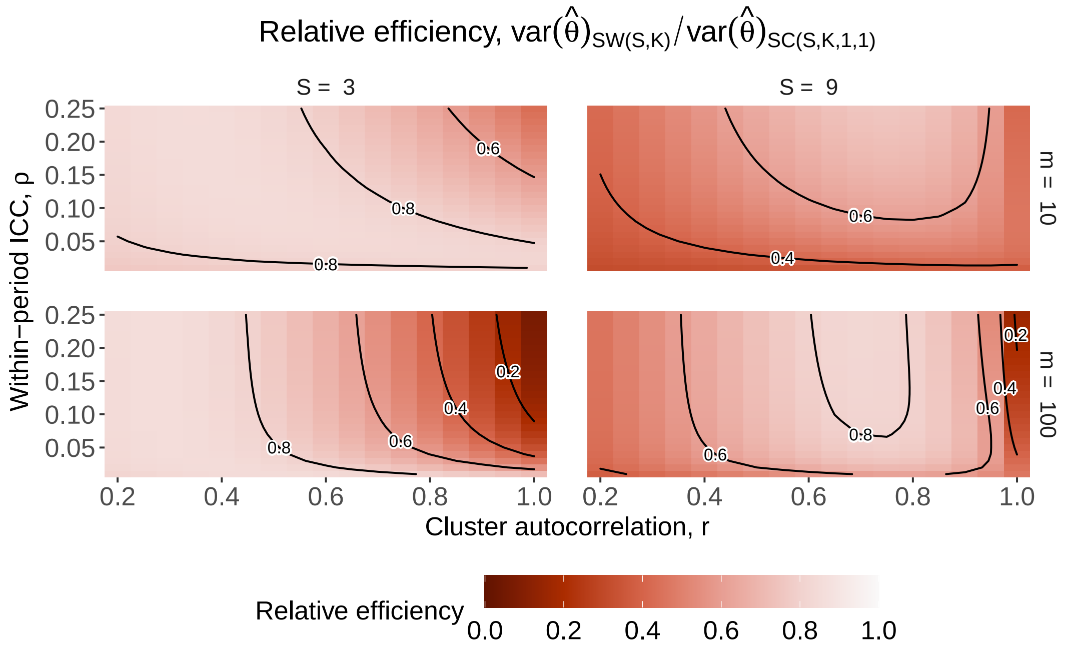

Figure S5: Relative efficiency for $SC(S, K, 1, 1)$ embedded basic staircase designs compared to $SW(S, K)$ stepped wedge designs, with $S = 3$ sequences (left column) and $S = 9$ sequences (right column), and with cluster-period sizes of $m = 10$ (top row) and $m = 100$ (bottom row), for the discrete-time decay correlation structure assuming categorical period effects.

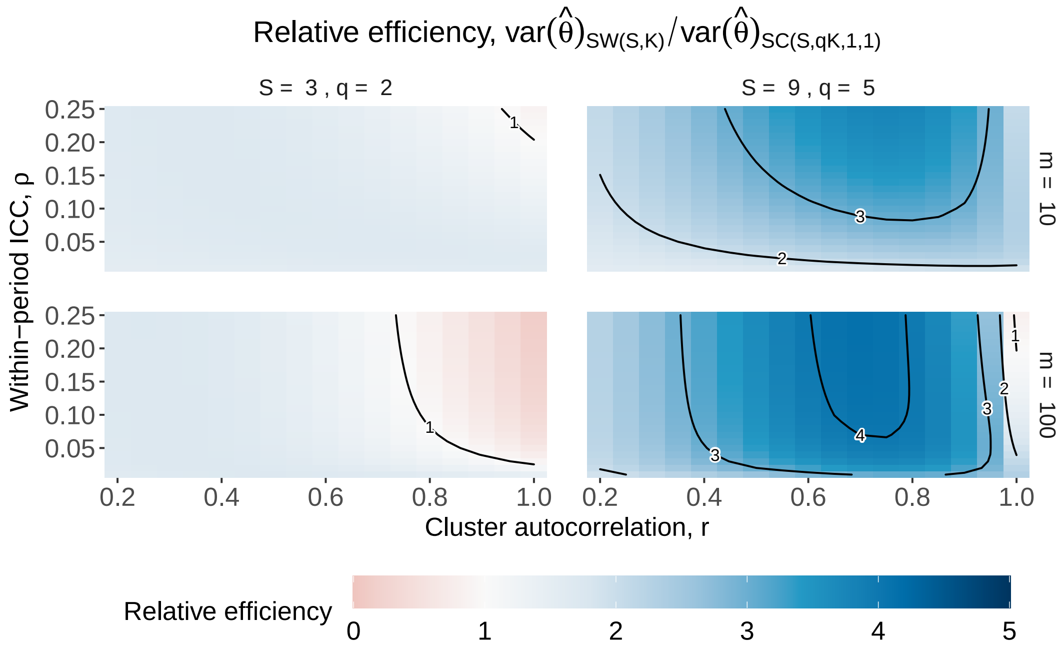

Figure S6: Relative efficiency for $SC(S, qK, 1, 1)$ extended staircase designs compared to $SW(S, K)$ stepped wedge designs, with $S = 3$ sequences and $q = 2$ (left column) and $S = 9$ sequences and $q = 5$ (right column), and with cluster-period sizes of $m = 10$ (top row) and $m = 100$ (bottom row), for the discrete-time decay correlation structure assuming categorical period effects.

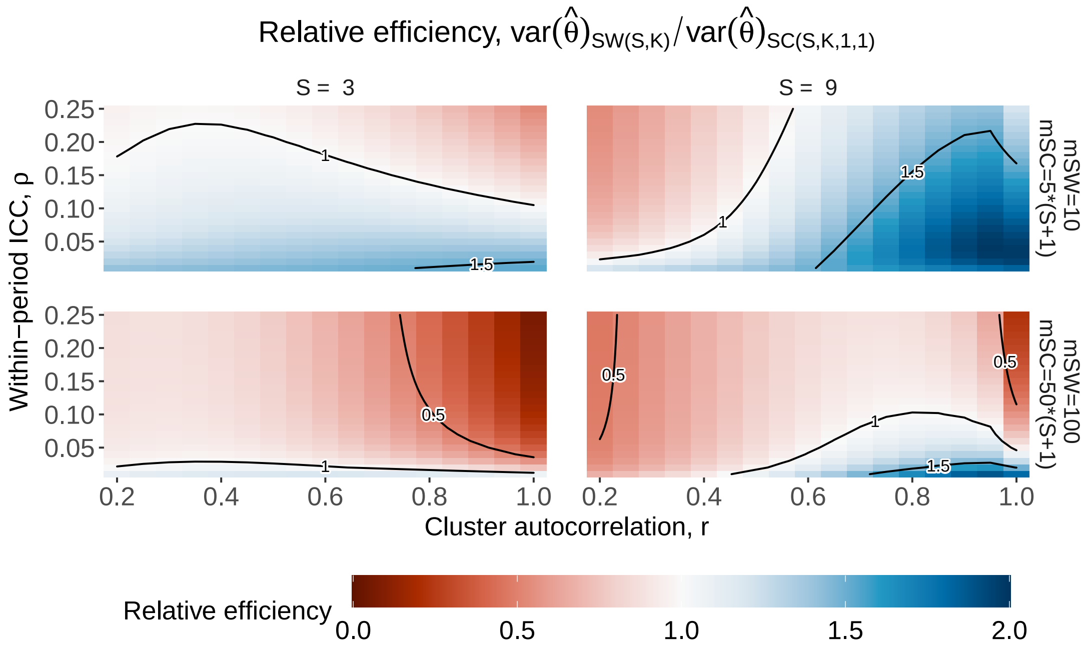

Figure S7: Relative efficiency for $SC(S, K, 1, 1)$ basic staircase designs compared to $SW(S, K)$ stepped wedge designs, with $S = 3$ sequences (left column) and $S = 9$ sequences (right column), and with cluster-period sizes of $m_{SW} = 10$ and $m_{SC} = 20$ (top-left), $m_{SW} = 10$ and $m_{SC} = 50$ (top-right), $m_{SW} = 100$ and $m_{SC} = 200$ (bottom-left), and $m_{SW} = 100$ and $m_{SC} = 500$ (bottom-right), for the discrete-time decay correlation structure assuming categorical period effects.

D.2: Assuming a linear effect of time

Were a linear effect of time assumed instead of categorical period effects, the patterns of relative efficiency are very similar (Figures S8-S10): in this case, only the variance for the staircase design is impacted (Grantham et al, 2024), while the variance for the stepped wedge design remains the same under categorical or linear period effects (Grantham et al, 2020). The variance for a basic staircase design is slightly lower under a linear time effect (Grantham et al, 2024), and so the relative efficiencies are all slightly higher than under categorical period effects.

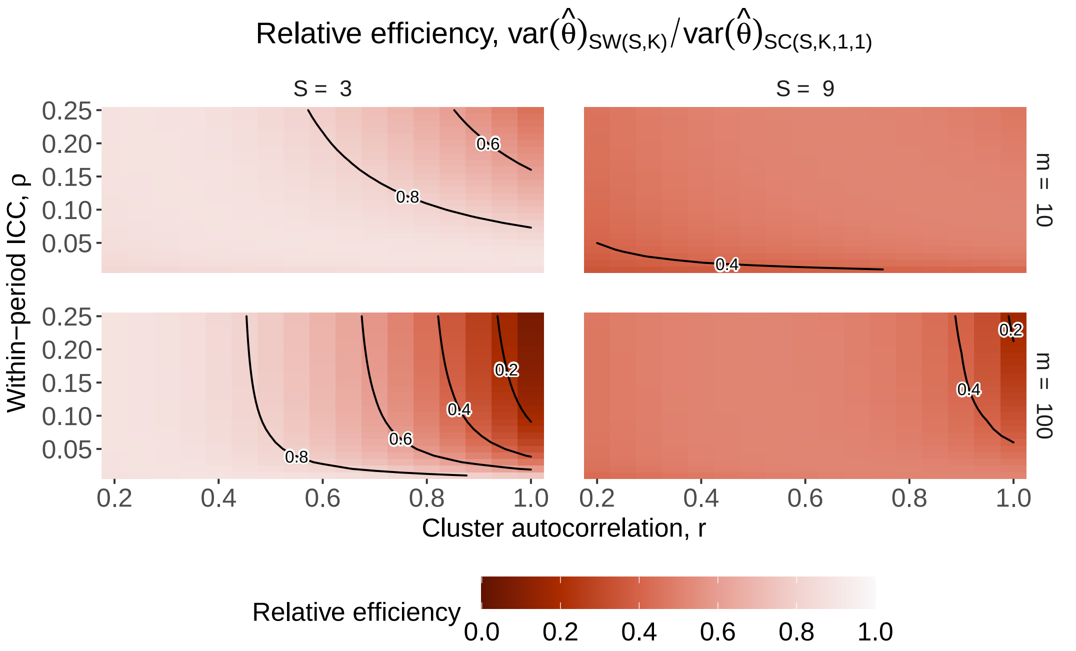

Figure S8: Relative efficiency for $SC(S,K,1,1)$ embedded basic staircase designs compared to $SW(S,K)$ stepped wedge designs, with $S = 3$ sequences (left column) and $S = 9$ sequences (right column), and with cluster-period sizes of $m = 10$ (top row) and $m = 100$ (bottom row), for the block-exchangeable intracluster correlation structure assuming linear period effects.

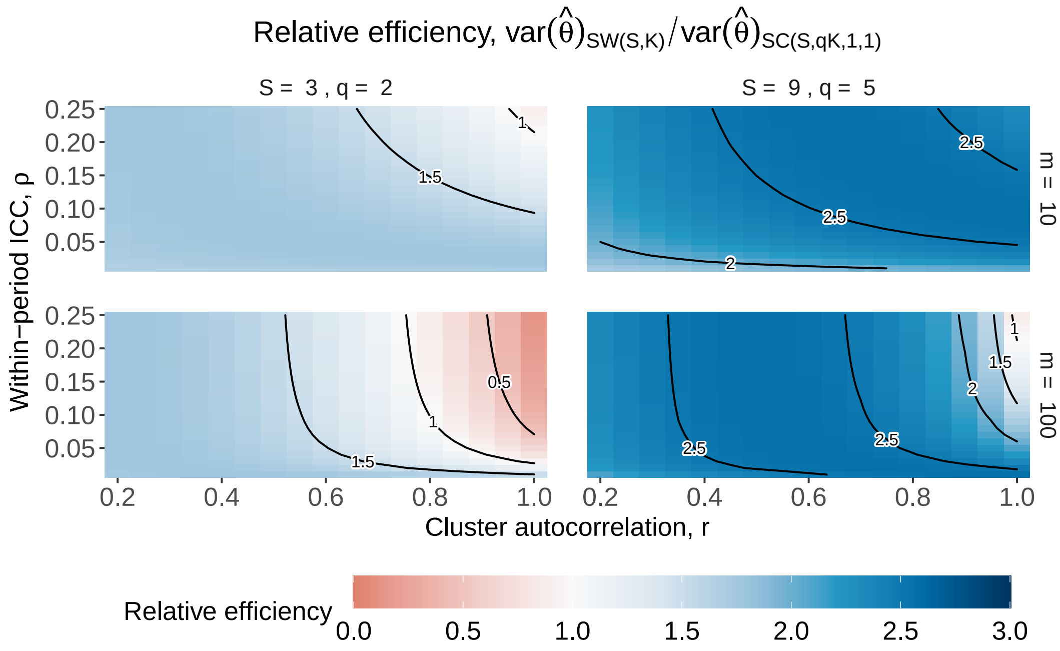

Figure S9: Relative efficiency for $SC(S, qK, 1, 1)$ extended staircase designs compared to $SW(S, K)$ stepped wedge designs, with $S = 3$ sequences and $q = 2$ (left column) and $S = 9$ sequences and $q = 5$ (right column), and with cluster-period sizes of $m = 10$ (top row) and $m = 100$ (bottom row), for the block-exchangeable intracluster correlation structure assuming linear period effects.

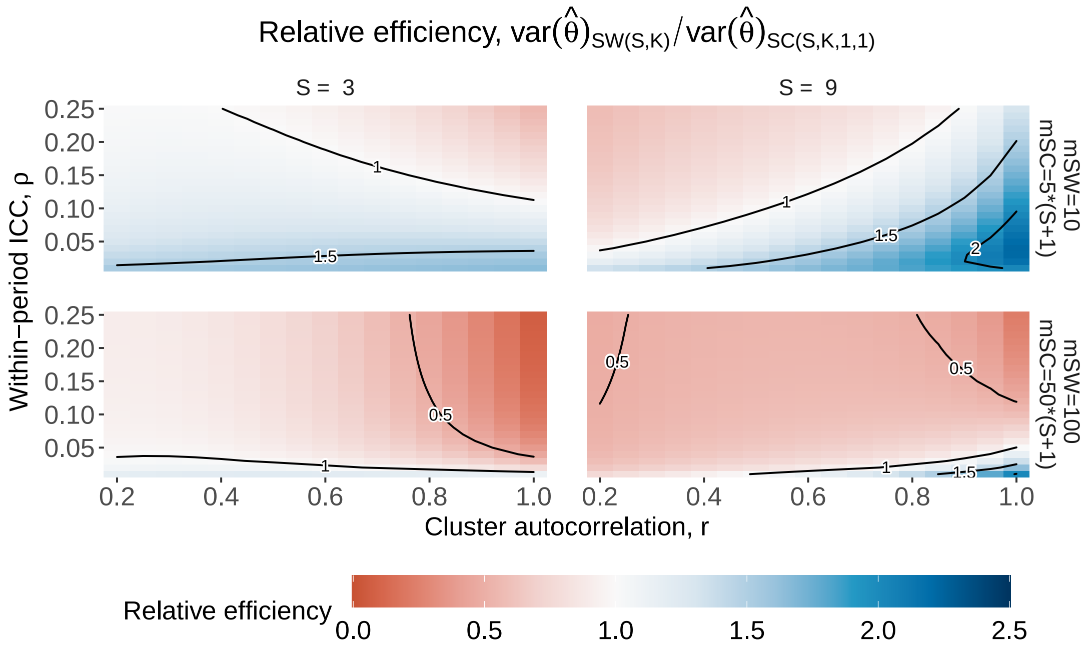

Figure S10: Relative efficiency for $SC(S, K, 1, 1)$ basic staircase designs compared to $SW(S, K)$ stepped wedge designs, with $S = 3$ sequences (left column) and $S = 9$ sequences (right column), and with cluster-period sizes of $m_{SW} = 10$ and $m_{SC} = 20$ (top-left), $m_{SW} = 10$ and $m_{SC} = 50$ (top-right), $m_{SW} = 100$ and $m_{SC} = 200$ (bottom-left), and $m_{SW} = 100$ and $m_{SC} = 500$ (bottom-right), for the block-exchangeable intracluster correlation structure assuming linear period effects.

E: Relative efficiency plots for alternative sampling schemes and design types

E.1: Trials with a cohort sampling scheme

Our results can be extended to closed cohort sampling schemes, in which the same set of participants within a cluster provides measurements in each of the trial periods, and also to open cohort sampling schemes in which some participants provide measurements in more than one period. Both of these extensions to our results are enabled with the necessary modifications to the correlation between cluster-period means, ψ. Models for stepped wedge and staircase designs with a closed cohort sampling scheme involve an additional within-participant correlation, e.g. ρ_u as in Grantham et al (2024). Again assuming a total variance of one, the definition of the correlation between cluster-period means changes slightly to $\psi = \frac{\rho_u + m\rho r}{1+(m-1)\rho}$, and so for closed cohort schemes where $\rho_u > 0$, the values of ψ for a particular combination of m, ρ and r are larger than for trials with a repeated cross-sectional sampling scheme where $\rho_u = 0$. However, the relationship between the relative efficiency and ψ remains the same: Figures 3 and 5 still apply to closed cohort schemes when stepped wedge designs are compared to embedded and extended basic staircase designs, respectively. Since the relative efficiencies are nonlinear functions of ψ, the magnitudes of the relative efficiencies for different combinations of m, ρ and r will be slightly higher or lower, while the patterns are generally similar to those seen in Sections 3.1 to 3.3 (see Figures S11-S16). For open cohort sampling schemes, ψ is defined similarly with the exception that ρ_u is multiplied by the retention rate, or the proportion of participants measured in one cluster-period who also provide measurements in the next period (Kasza et al, 2020). Figures 3 and 5 again apply to open cohort schemes, but since the retention rate(s) will be less than one, the values of ψ for a particular combination of m, ρ and r will be smaller than for closed cohort trials (where the retention rate is equal to one) but larger than for trials with a repeated cross-sectional sampling scheme; the patterns of the relative efficiencies will again be similar, while the magnitudes of the relative efficiencies will differ slightly from those of the other sampling schemes.

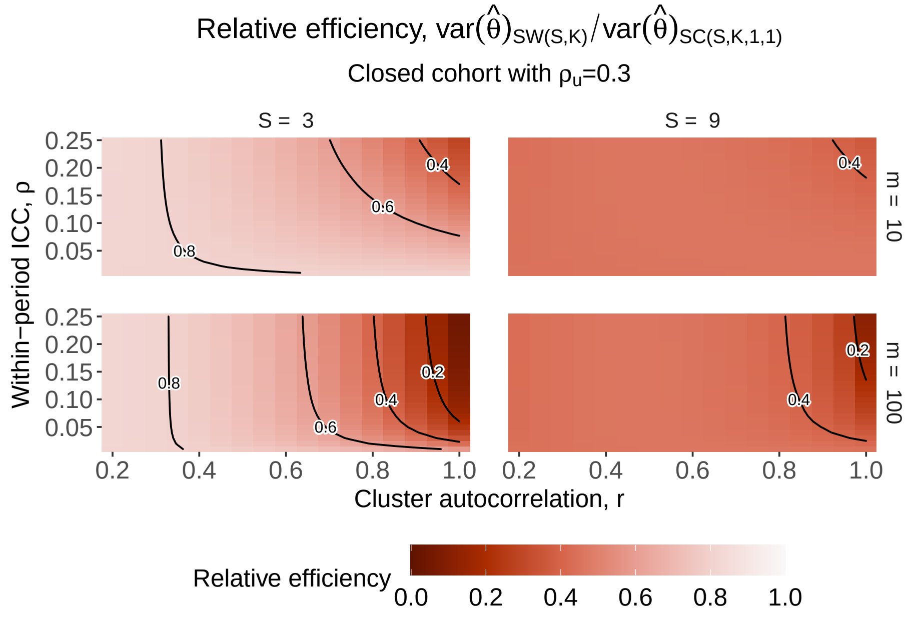

Figure S11: Relative efficiency for $SC(S, K, 1, 1)$ embedded basic staircase designs compared to $SW(S, K)$ stepped wedge designs, with $S = 3$ sequences (left column) and $S = 9$ sequences (right column), and with cluster-period sizes of $m = 10$ (top row) and $m = 100$ (bottom row), for closed cohort designs with within-participant correlation of $\rho_u = 0.3$, for the block-exchangeable correlation structure assuming categorical period effects.

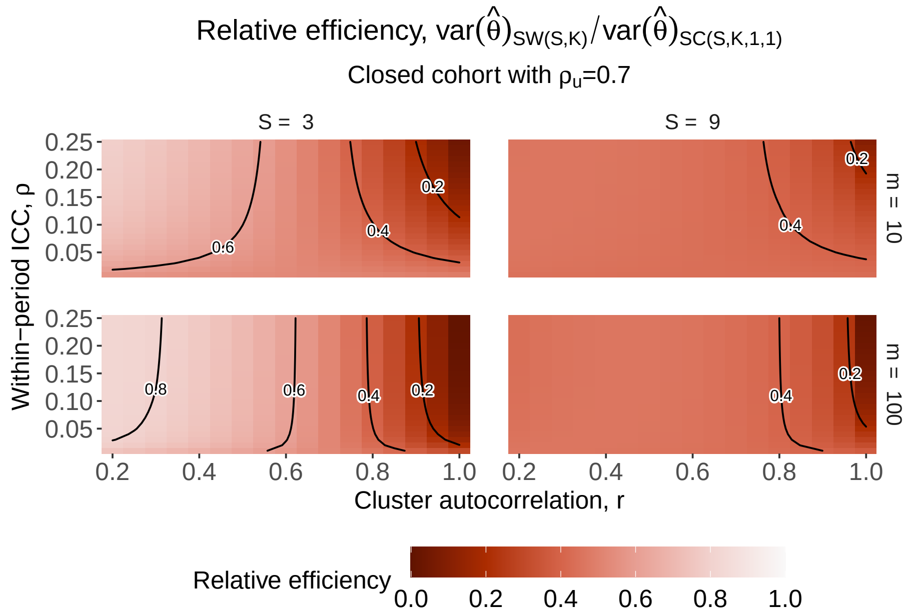

Figure S12: Relative efficiency for $SC(S, K, 1, 1)$ embedded basic staircase designs compared to $SW(S, K)$ stepped wedge designs, with $S = 3$ sequences (left column) and $S = 9$ sequences (right column), and with cluster-period sizes of $m = 10$ (top row) and $m = 100$ (bottom row), for closed cohort designs with within-participant correlation of $\rho_u = 0.7$, for the block-exchangeable correlation structure assuming categorical period effects.

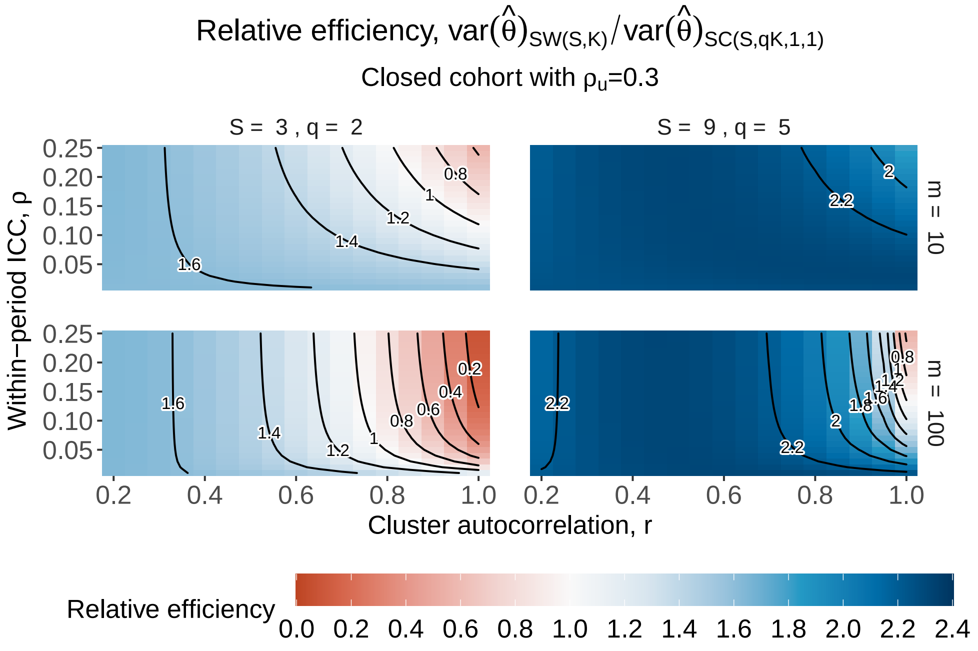

Figure S13: Relative efficiency for $SC(S, qK, 1, 1)$ extended staircase designs compared to $SW(S, K)$ stepped wedge designs, with $S = 3$ sequences and $q = 2$ (left column) and $S = 9$ sequences and $q = 5$ (right column), and with cluster-period sizes of $m = 10$ (top row) and $m = 100$ (bottom row), for closed cohort designs with within-participant correlation of $\rho_u = 0.3$, for the block-exchangeable intracluster correlation structure assuming categorical period effects.

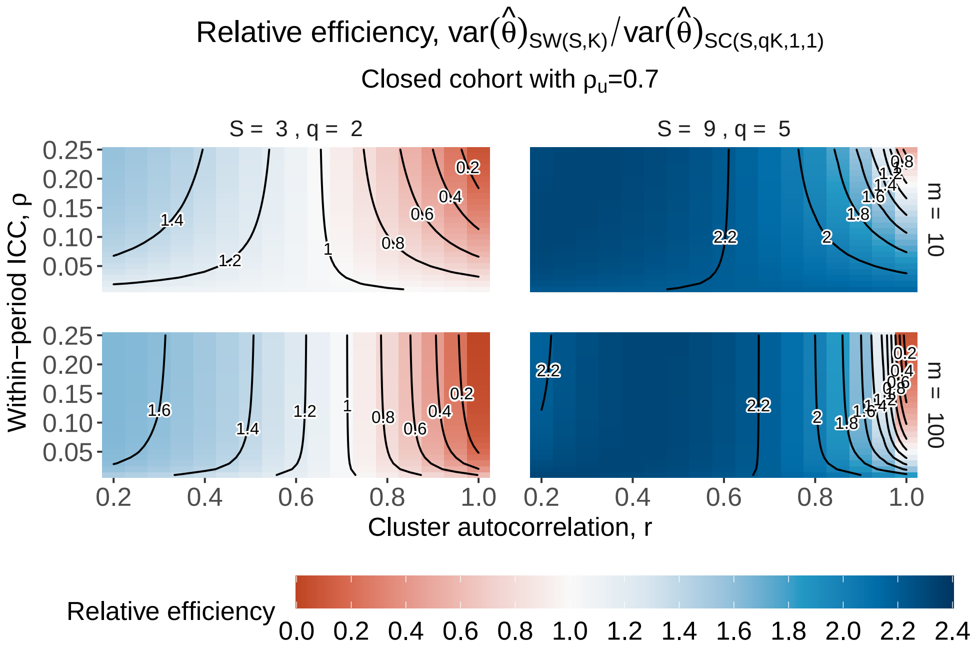

Figure S14: Relative efficiency for $SC(S, qK, 1, 1)$ extended staircase designs compared to $SW(S, K)$ stepped wedge designs, with $S = 3$ sequences and $q = 2$ (left column) and $S = 9$ sequences and $q = 5$ (right column), and with cluster-period sizes of $m = 10$ (top row) and $m = 100$ (bottom row), for closed cohort designs with within-participant correlation of $\rho_u = 0.7$, for the block-exchangeable intracluster correlation structure assuming categorical period effects.

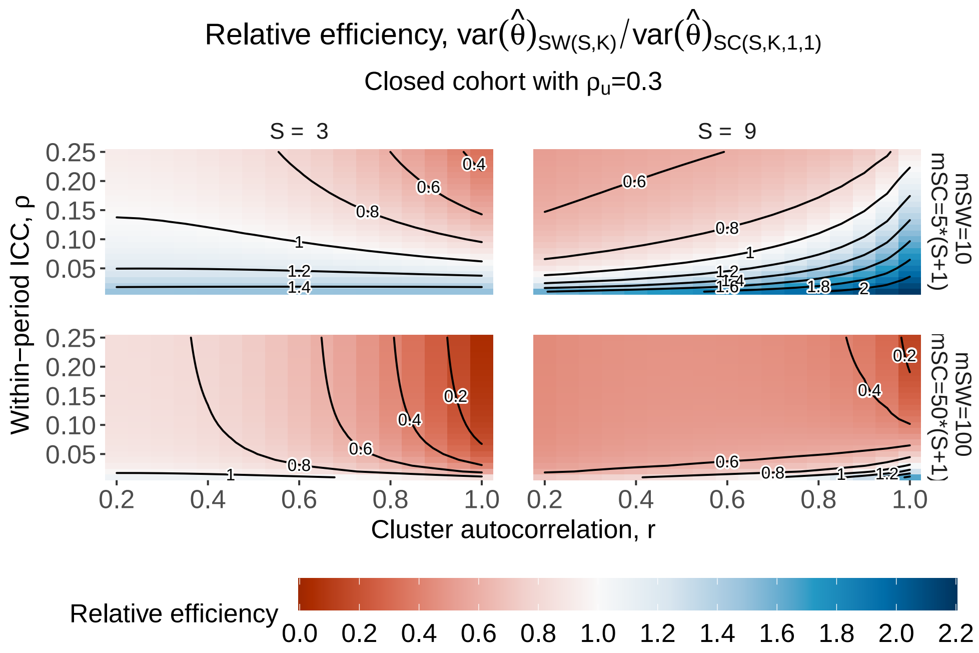

Figure S15: Relative efficiency for $SC(S, K, 1, 1)$ basic staircase designs compared to $SW(S, K)$ stepped wedge designs, with $S = 3$ sequences (left column) and $S = 9$ sequences (right column), and with cluster-period sizes of $m_{SW} = 10$ and $m_{SC} = 20$ (top-left), $m_{SW} = 10$ and $m_{SC} = 50$ (top-right), $m_{SW} = 100$ and $m_{SC} = 200$ (bottom-left), and $m_{SW} = 100$ and $m_{SC} = 500$ (bottom-right), for closed cohort designs with within-participant correlation of $\rho_u = 0.3$, for the block-exchangeable intracluster correlation structure assuming categorical period effects.

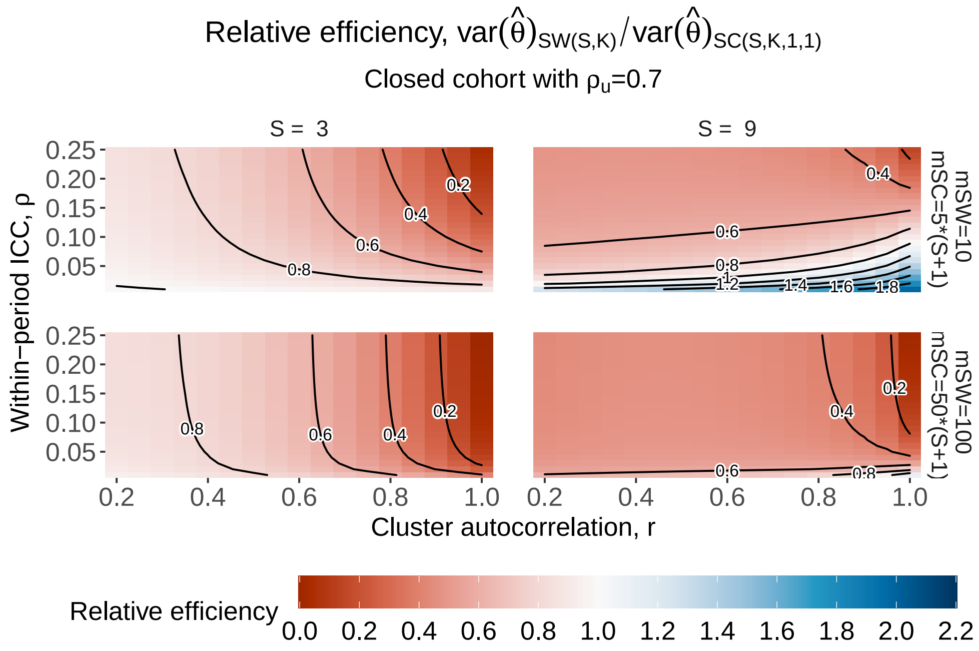

Figure S16: Relative efficiency for $SC(S,K,1,1)$ basic staircase designs compared to $SW(S,K)$ stepped wedge designs, with $S = 3$ sequences (left column) and $S = 9$ sequences (right column), and with cluster-period sizes of $m_{SW} = 10$ and $m_{SC} = 20$ (top-left), $m_{SW} = 10$ and $m_{SC} = 50$ (top-right), $m_{SW} = 100$ and $m_{SC} = 200$ (bottom-left), and $m_{SW} = 100$ and $m_{SC} = 500$ (bottom-right), for closed cohort designs with within-participant correlation of $\rho_u = 0.7$, for the block-exchangeable intracluster correlation structure assuming categorical period effects.

E.2: Designs with implementation periods

Implementation, or transition, periods may be included after the final control period and before the first intervention period to allow time for the intervention to be introduced before measuring participants under the intervention condition (Figure S17 shows schematics for nine-sequence designs). Note that we would not recommend three-sequence basic staircase designs with implementation periods as this design provides minimal overlap within periods, and the treatment effect estimator relies on within-period comparisons. The patterns of relative efficiencies for designs including implementation periods are broadly similar to those for designs without implementation periods (see Figures S18-S20). Both the stepped wedge and staircase designs have lower precision when implementation periods are included, but the relative efficiencies for particular combinations of correlation values may be slightly higher or lower than for the designs without implementation periods.

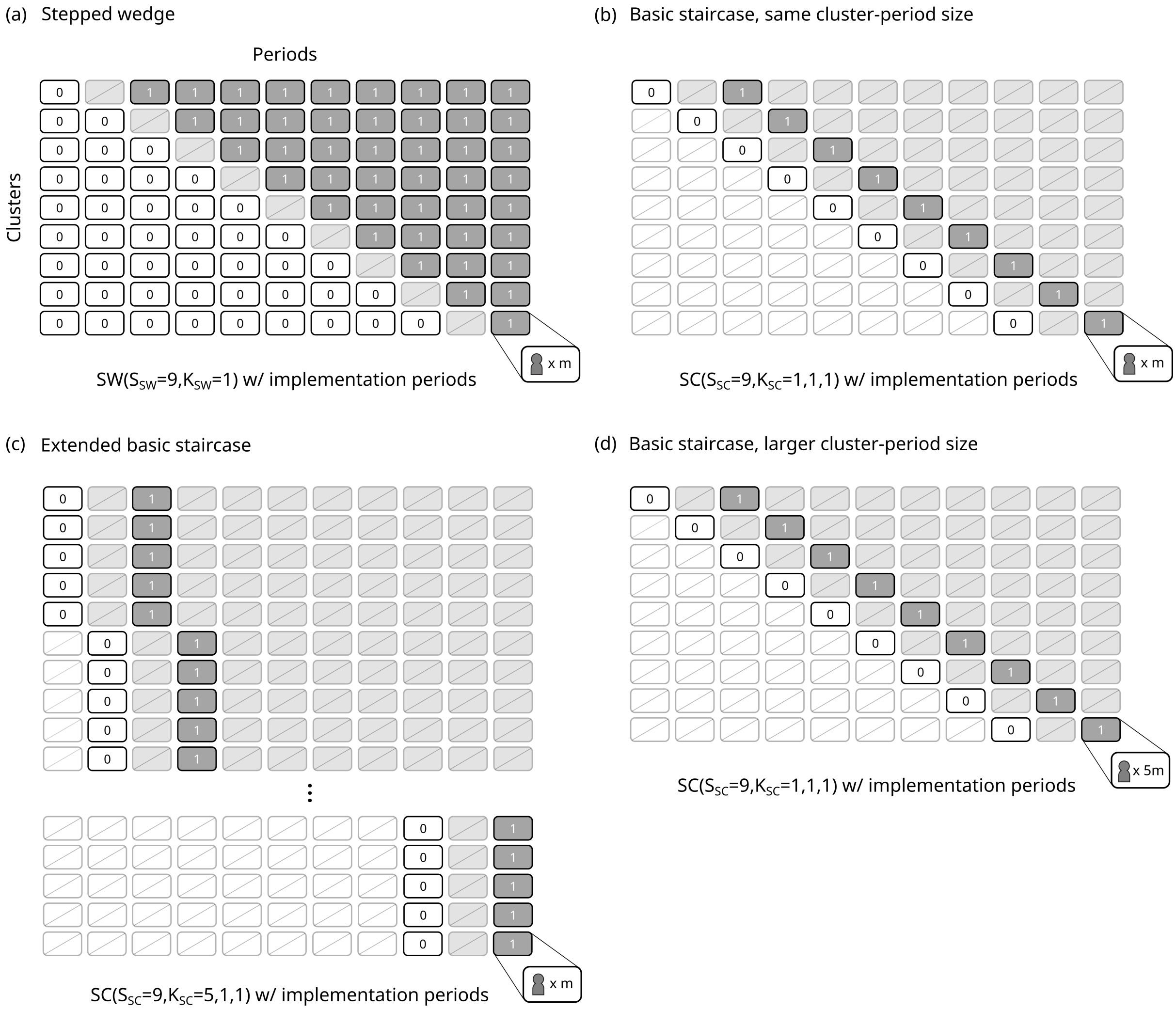

Figure S17: Design schematics for the nine-sequence designs with implementation periods to be compared, shown with a fixed number of clusters per sequence for illustrative purposes: (a) a stepped wedge design with implementation periods, one cluster per sequence and a cluster-period size of m, (b) a basic staircase design with implementation periods, one cluster per sequence and a cluster-period size of m, (c) an extended basic staircase design with implementation periods, five clusters per sequence and a cluster-period size of m, and (d) a basic staircase design with implementation periods, one cluster per sequence and a larger cluster-period size of $5m$.

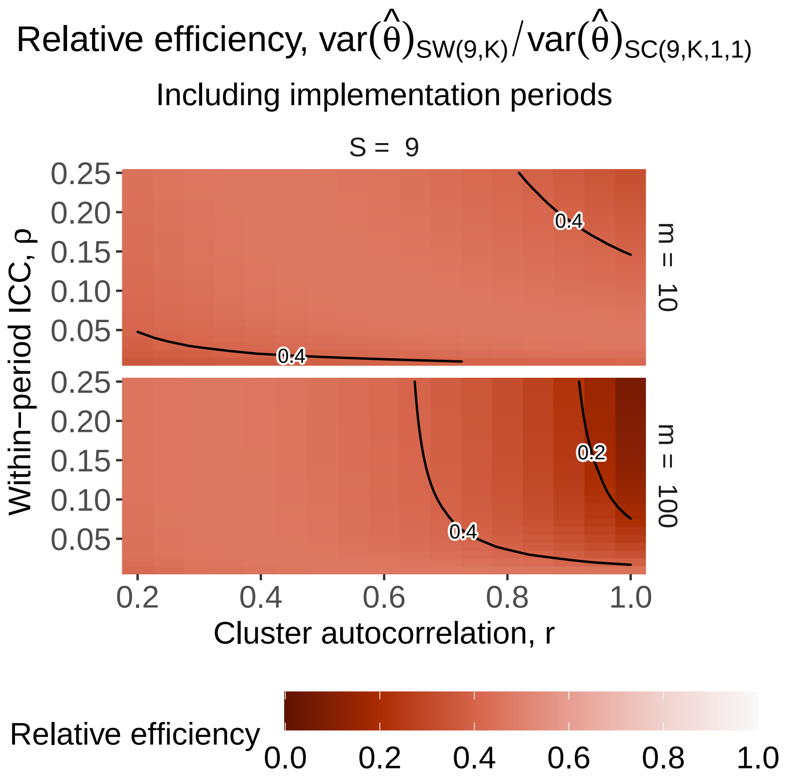

Figure S18: Relative efficiency for $SC(9, K, 1, 1)$ embedded basic staircase designs with implementation periods compared to $SW(9, K)$ stepped wedge designs with implementation periods, with cluster-period sizes of $m = 10$ (top row) and $m = 100$ (bottom row), for the block-exchangeable correlation structure assuming categorical period effects.

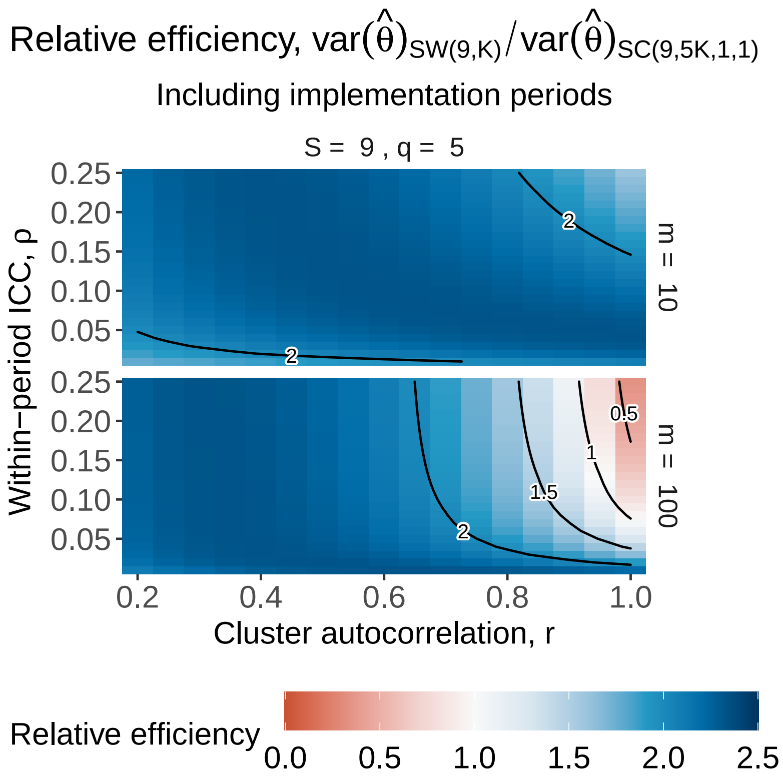

Figure S19: Relative efficiency for $SC(9, 5K, 1, 1)$ extended staircase designs with implementation periods compared to $SW(9, K)$ stepped wedge designs with implementation periods, with cluster-period sizes of $m = 10$ (top row) and $m = 100$ (bottom row), for the block-exchangeable intracluster correlation structure assuming categorical period effects.

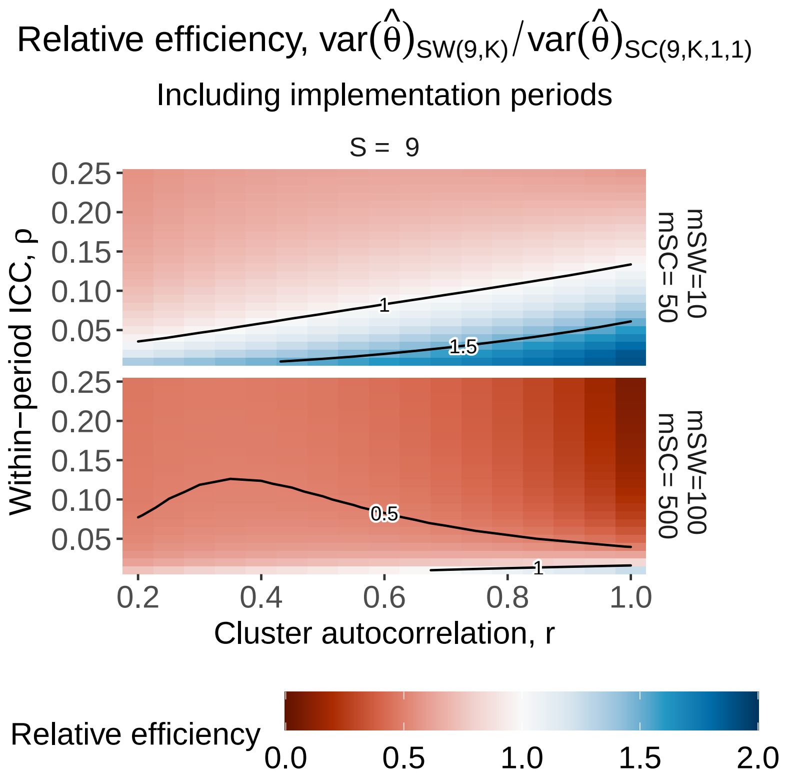

Figure S20: Relative efficiency for $SC(9, K, 1, 1)$ basic staircase designs with implementation periods compared to $SW(9, K)$ stepped wedge designs with implementation periods, with cluster-period sizes of $m_{SW} = 10$ and $m_{SC} = 50$ (top) and $m_{SW} = 100$ and $m_{SC} = 500$ (bottom), for the block-exchangeable intracluster correlation structure assuming categorical period effects.

References

Grantham KL, Forbes AB, Heritier S, Kasza J. Time parameterizations in cluster randomized trial planning. *Am Stat* 2020; 74(2): 184-189.

Li F, Forbes AB, Turner EL, Preisser JS. Power and sample size requirements for GEE analyses of cluster randomized crossover trials. *Stat Med* 2019; 38(4): 636-649.

Graybill FA. *Matrices with Applications in Statistics.* Belmont, CA: Brooks/Cole; 1983.

Hussey MA and Hughes JP. Design and analysis of stepped wedge cluster randomized trials. *Contemp Clin Trials* 2007; 28(2): 182-191.

Grantham KL, Forbes AB, Hooper R, Kasza J. The staircase cluster randomised trial design: A pragmatic alternative to the stepped wedge. *Stat Methods Med Res* 2024; 33(1): 24-41.

Kasza J, Hooper R, Copas A, Forbes AB. Sample size and power calculations for open cohort longitudinal cluster randomized trials. *Stat Med* 2020; 39(13): 1871-1883.
